# Metatranscriptome Reveals Specific Immune and Microbial Signatures of Respiratory Syncytial Virus Infection in Children

**DOI:** 10.1128/spectrum.04107-22

**Published:** 2023-03-02

**Authors:** Qianyu Feng, Ziheng Feng, Bin Yang, Shuaibing Han, Shunhang Wen, Gen Lu, Rong Jin, Baoping Xu, Hailin Zhang, Lili Xu, Zhengde Xie

**Affiliations:** a Beijing Key Laboratory of Pediatric Respiratory Infection Diseases, Key Laboratory of Major Diseases in Children, Ministry of Education, National Clinical Research Center for Respiratory Diseases, National Key Discipline of Pediatrics (Capital Medical University), Beijing Pediatric Research Institute, Beijing Children’s Hospital, Capital Medical University, National Center for Children’s Health, Beijing, China; b Research Unit of Critical Infection in Children, Chinese Academy of Medical Sciences, Beijing, China; c Vision Medicals Center for Infectious Diseases, Guangzhou, Guangdong, China; d Department of Children’s Respiration disease, the Second Affiliated Hospital & Yuying Children's Hospital, Wenzhou Medical University, Wenzhou, Zhejiang, China; e Guiyang Women and Children Healthcare Hospital, Guiyang, Guizhou, China; f Department of Respiratory Diseases I, Beijing Children’s Hospital, Capital Medical University, National Clinical Research Center for Respiratory Diseases, National Center for Children’s Health, Beijing, China; Shandong First Medical University

**Keywords:** host expression profile, respiratory syncytial virus, adenovirus, influenza virus, human metapneumovirus, microbiome

## Abstract

Respiratory syncytial virus (RSV) is the most frequently detected respiratory virus in children with acute lower respiratory tract infection. Previous transcriptome studies have focused on systemic transcriptional profiles in blood and have not compared the expression of multiple viral transcriptomes. Here, we sought to compare transcriptome responses to infection with four common respiratory viruses for children (respiratory syncytial virus, adenovirus, influenza virus, and human metapneumovirus) in respiratory samples. Transcriptomic analysis showed that cilium organization and assembly were common pathways related to viral infection. Compared with other virus infections, collagen generation pathways were distinctively enriched in RSV infection. We identified two interferon-stimulated genes (ISGs), *CXCL11* and *IDO1*, which were upregulated to a greater extent in the RSV group. In addition, a deconvolution algorithm was used to analyze the composition of immune cells in respiratory tract samples. The proportions of dendritic cells and neutrophils in the RSV group were significantly higher than those in the other virus groups. The RSV group exhibited a higher richness of Streptococcus than the other virus groups. The concordant and discordant responses mapped out here provide a window to explore the pathophysiology of the host response to RSV. Last, according to host-microbe network interference, RSV may disrupt respiratory microbial composition by changing the immune microenvironment.

**IMPORTANCE** In the present study, we demonstrated the comparative results of host responses to infection between RSV and other three common respiratory viruses for children. The comparative transcriptomics study of respiratory samples sheds light on the significant roles that ciliary organization and assembly, extracellular matrix changes, and microbial interactions play in the pathogenesis of RSV infection. Additionally, it was demonstrated that the recruitment of neutrophils and dendritic cells (DCs) in the respiratory tract is more substantial in RSV infection than in other viral infections. Finally, we discovered that RSV infection dramatically increased the expression of two ISGs (*CXCL11* and *IDO1*) and the abundance of Streptococcus.

## INTRODUCTION

Respiratory syncytial virus (RSV) is the most important viral pathogen causing acute lower respiratory tract infection (ALRTI) in children less than 5 years old worldwide (1), posing huge medical and economic burdens globally (2). However, there is currently no effective anti-RSV medicine or vaccine, and clinical practice is restricted to supportive care (3). Therefore, aiming to reveal new therapeutic targets and help with vaccine research and development, insight into the pathophysiology of RSV infection is needed.

The interaction between viruses and hosts is crucial in the pathogenesis of viral infection. The molecular mechanisms associated with specific diseases and individual responses to infection can be understood and described through transcriptomics (4). There are currently a few studies about host expression profiles for RSV infection, but most of them are limited to systemic transcriptional profiles in blood (5–7). Although peripheral blood mononuclear cells are involved in innate immune responses to infection, they are not directly infected by RSV and are unable to reflect immune cell composition changes in the airway, which is the primary infection site of RSV (8). Therefore, analysis of the transcriptome of respiratory specimens has the potential to gain valuable insights into the pathogenesis of RSV.

In addition to host-virus interactions, the host microbiome composition at the infection site frequently influences the host response against viruses indirectly and the overall risk of respiratory tract infection development (9). There are reports about commensal and pathogenic microbial disruption after RSV infection (7, 10), but few studies have reported comparisons of microbial composition between respiratory samples from patients with RSV and those with other common respiratory viruses.

In this work, transcriptome sequencing (RNA-seq) was applied to profile the transcriptome and microbiome of respiratory samples from 25 patients with RSV infection, 52 patients with other virus infections, and 15 healthy children. Here, we report comparative results of host responses to infection between RSV and other viruses. We mapped their similarities and differences at the gene level, pathway level, and cell proportion level to gain insight into the specific immune signatures of RSV infection. In addition, we analyzed the active microbiome of three different groups and identified specific biomarkers of different groups. Host-microbe network interference was built to explore the relationship between the host immune response and microorganisms during RSV and other virus infections.

## RESULTS

### Host gene expression profiles among the RSV group, non-RSV group, and control group.

**FIG 1:**
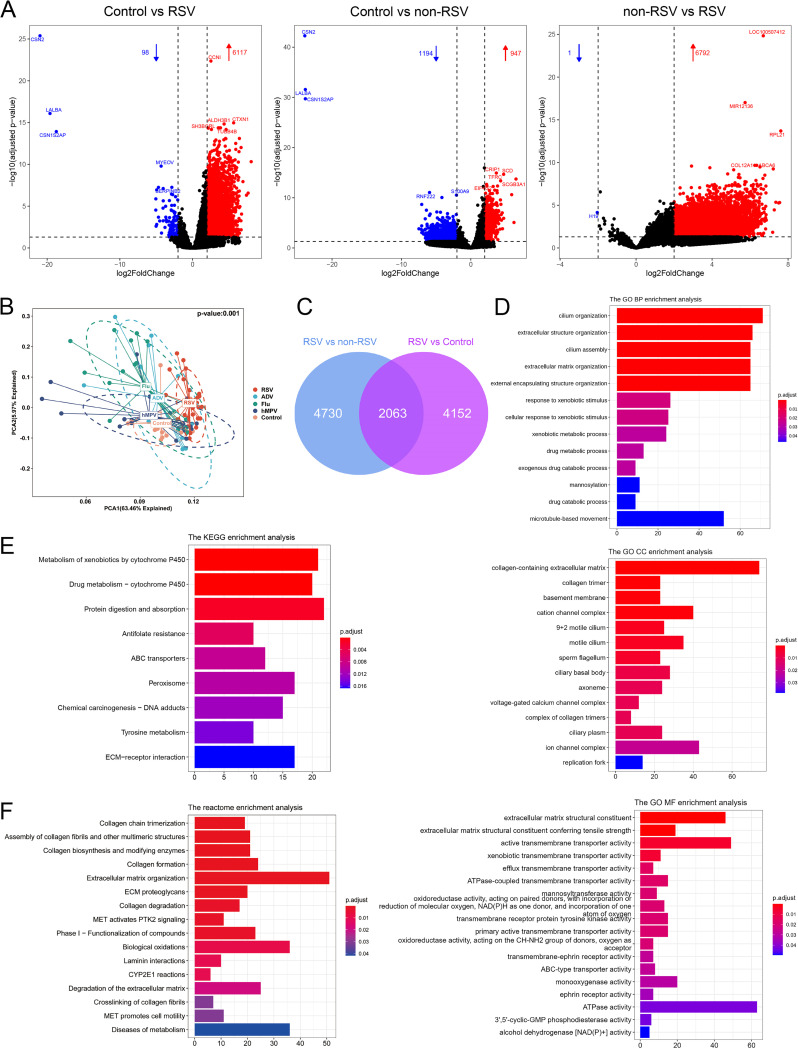
RNA-seq data for patients with RSV versus controls and pathway analysis of the RSV signature. (A) Volcano plots showing DEGs in control versus RSV, control versus non-RSV, and non-RSV versus RSV. Upregulated genes (red) and downregulated genes (blue) are marked. (B) PCoA of global gene expression among different viral infection groups and control groups. (C) Venn diagram showing DEGs in RSV versus non-RSV (left circle) and RSV versus control (right circle). A total of 2,063 genes differentially expressed in RSV versus control and RSV versus non-RSV represented RSV-specific gene signatures. (D to F) GO term enrichment analysis of biological processes (BP), cellular component (CC), molecular function (MF) (D), KEGG (E), and Reactome database (F). The gene ratio (*x* axis) is the ratio of the number of genes in our data enriched in a given gene set (pathway) to the total number of genes in that pathway.

First, we analyzed whether the transcriptome results of different sample types were highly heterogeneous before comparing the transcriptome of different viruses. Principal-coordinate analysis (PCoA) of RNA expression between 25 sputum samples (SPs) and 53 nasopharyngeal swabs (NPs) showed there was no significant heterogeneity between the nasopharyngeal swab and the airway aspirate (see Fig. S1 in the supplemental material). We enrolled and sequenced 25 and 15 airway aspirations from pediatric patients with RSV infection (defined as the RSV group) and healthy children (defined as the control group), respectively. RNA-seq identified 6,215 differentially expressed genes (DEGs; 6,117 overexpressed, 98 underexpressed) with |log_2_FC| of ≥2 and adjusted *P* value of <0.05, referred to as the “RSV signature” (Fig. 1A). In addition, the differential expression analysis of the metatranscriptome from 52 patients with other viruses (18 patients with influenza virus [flu], 13 patients with adenovirus [AdV], and 21 patients with human metapneumovirus [hMPV], defined as the non-RSV group) identified 2,141 DEGs (947 overexpressed, 1,194 underexpressed) compared to the control group, referred to as the “non-RSV signature” (Fig. 1A). The PCoA of the global expression profile demonstrated that there were substantial differences among the groups (Fig. 1B). In addition, to identify a statistically significant set of genes differentially expressed in patients with RSV compared with those with other viral infections, we compared 25 patients with RSV versus 52 patients with non-RSV viral infections. A total of 6,793 DEGs were screened with |log_2_FC| of ≥2 and adjusted *P* value of <0.05 (Fig. 1A). By comparing these DEGs with the RSV signature, 2,063 genes overlapped and were referred to as the RSV-specific gene signature (Fig. 1C). For pathway analysis, the above-described RSV-specific gene signature DEGs were subjected to gene ontology (GO), Kyoto Encyclopedia of Genes and Genomes (KEGG), and Reactome pathway enrichment analysis. GO enrichment analysis identified a set of biological processes (BPs) associated with cilium biological processes, such as cilium organization and assembly, and with extracellular component activity, such as extracellular structure and extracellular matrix (ECM) organization (Fig. 1D); a set of cellular components (CCs), such as collagen-containing extracellular matrix, ion channel complex, and cation channel complex (Fig. 1D); and a set of molecular functions (MFs), such as ATPase activity, active transmembrane transporter activity, and extracellular matrix structural constituents (Fig. 1D). Meanwhile, KEGG analysis highlighted the involvement of protein digestion and absorption, metabolism of xenobiotics by cytochrome P450, drug metabolism-cytochrome P450, etc. (Fig. 1E). Moreover, Reactome pathway analysis of this gene set identified collagen generation and assembly along with extracellular matrix as the top Reactome gene ontology terms (Fig. 1F).

### Differential immune gene expression and immune cell infiltration between the RSV and non-RSV groups.

**FIG 2:**
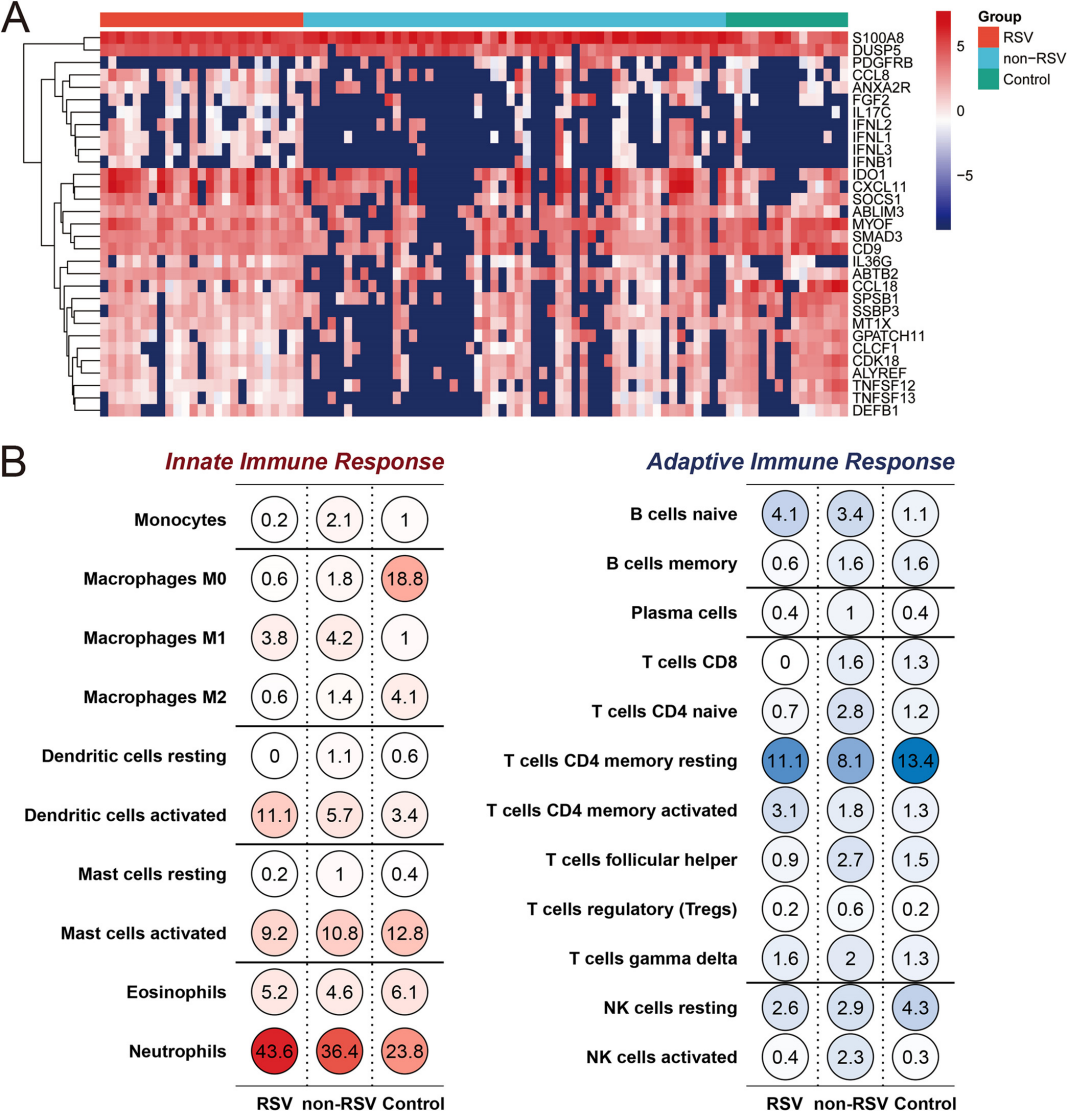
(A) Heatmap for differentially expressed immune-related genes and interferon-stimulated genes (ISGs) with *P* values of <0.05 in the RSV group versus the control group and non-RSV group. (B) Changes in cell proportions when comparing patients with RSV to non-RSV and controls. Note the trends of increased dendritic cells and neutrophils in RSV infection.

Next, to explore the difference in immune responses against viruses between the RSV group and non-RSV group, immune-related genes (IRGs) were screened by *P* values of <0.05 in the RSV group compared with the control and non-RSV groups. A total of 14 genes were found to be significantly differentially expressed in the RSV group compared with both the non-RSV and control groups (Table 1). The heatmap showed that the expression of *CCL8*, *IL36G*, *FGF2*, *IL17C*, *IFNL2*, *IFNL1*, *IFNL3*, and *IFNB1* was low in the non-RSV group, while their expression decreased in only a few samples in the RSV group (Fig. 2A; Fig. S2). In addition, the expression levels of *CXCL11* and *IL36G* in the RSV group were remarkably higher than those in the non-RSV and control groups; however, the expression of some IRGs, such as *TNFSF12*, *TNFSF13*, and *IFNL1*, in the RSV group showed the opposite trend (Fig. 2A, Fig. S2, and Table 1).

**TABLE 1:** Immune-related genes with significantly different expression (*P* < 0.05) comparing RSV with non-RSV or controls

Gene	Control vs RSV *P* value	Non-RSV vs RSV *P* value
*IFNL3*	0.00034	0.00003
*IFNB1*	0.013	0.021
*CCL18*	0.0117	0.04926
*CXCL11*	0.000024	0.0048
*TNFSF13*	0.0136	0.0336
*CCL8*	0.0016	0.0315
*IFNL2*	0.0021	0.01
*TNFSF12*	0.00563	0.01841
*IFNL1*	0.00231	0.00043
*PDGFRB*	0.034	0.017
*IL36G*	0.0016	0.0014
*FGF2*	0.02	0.042
*IL17C*	0.044	0.014
*CLCF1*	0.0008	0.0381

**FIG 3:**
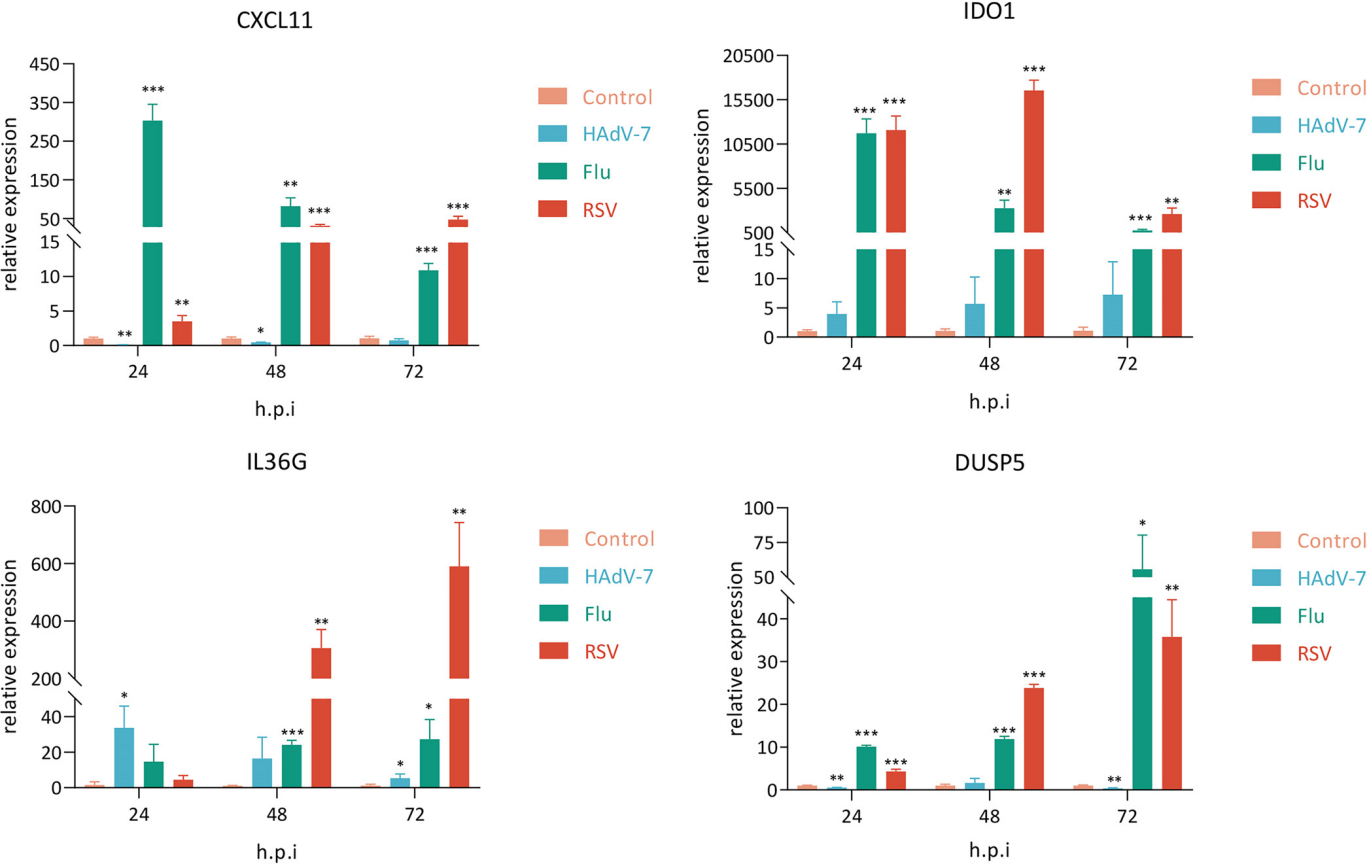
Real-time PCR quantification of selected immune-related genes in infected and control cells at 24, 48, and 72 h postinfection. RSV strains A2, HAdV-7, and PR8 were propagated in A549 cells with a multiplicity of infection (MOI) of 0.1. Statistical analysis was performed with unpaired *t* test. *P* values of <0.05 (*), <0.01 (**), and <0.001 (***) were considered significant. Bars in the figure panels show means and standard errors of the means (SEMs).

**FIG 4:**
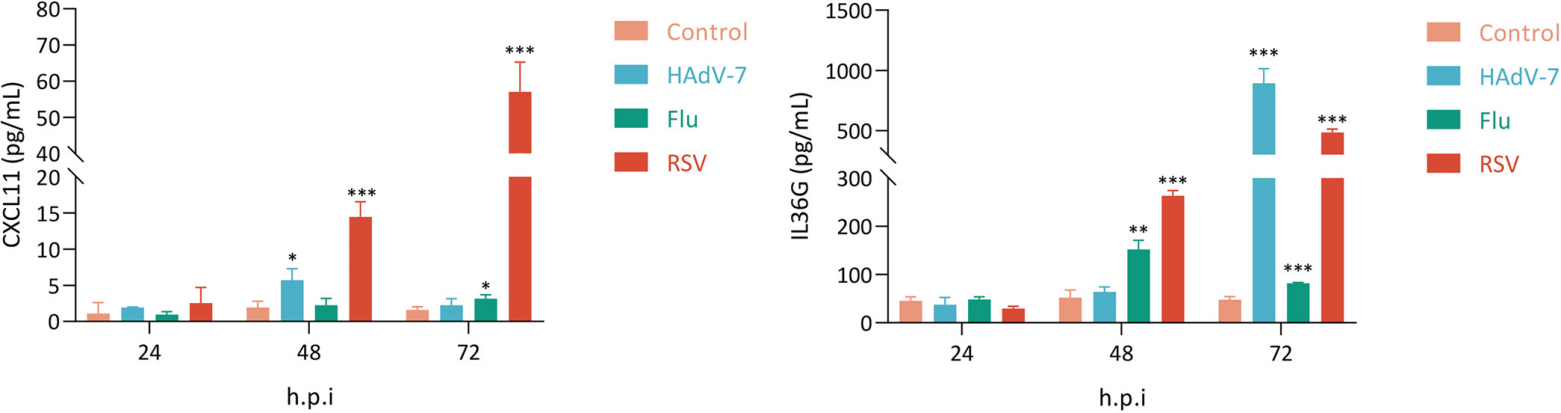
Cell-free supernatants were analyzed for CXCL11 (left) and IL-36G (right) secretion by ELISA. Statistical analysis was performed unpaired *t* test. *P* values of <0.05 (*), <0.01 (**), and <0.001 (***) were considered significant. Bars in the figure panels show means and standard errors of the means (SEM).

The expression of interferon-stimulated genes (ISGs) between the RSV group and non-RSV group or control group was also compared, and a total of 19 ISGs were screened out (Table 2) with *P* values of <0.05. *S100A8* and *DUSP5* exhibited high expression levels in all three groups (Fig. 2A; Fig. S2). The expression levels of *CCL8*, *DEFB1*, *ANXA2R*, *GPATCH11*, *SPSB1*, *MTX1*, *SSBP3*, *CDK18*, and *ALYREF* were downregulated in the non-RSV group, while their levels showed an upregulated pattern in the RSV group. The expression levels of some ISGs, such as *ABTB2*, *ABLIM3*, *CXCL11*, *SOCS1*, and *MYOF*, were partially downregulated in the non-RSV group but upregulated in almost all samples of the RSV group (Fig. 2A; Fig. S2). In addition, the expression levels of *CXCL11* and *IDO1* showed considerably increased patterns in the RSV group compared with both the control and non-RSV groups (Table 1; Fig. S2). Further validation by quantitative PCR (qPCR) showed that transcription levels of *CXCL11* and *IDO1* were significantly increased after RSV and flu infection of A549 cells (Fig. 3), but the *CXCL11* level in supernatant was only significantly increased after RSV infection (Fig. 4).

**TABLE 2:** Interferon-stimulated genes with significantly different expression (*P* < 0.05) comparing RSV with non-RSV or controls

Gene	Control vs RSV *P* value	Non-RSV vs RSV *P* value
*SSBP3*	0.0008	0.02292
*SPSB1*	0.000038	0.039
*CDK18*	0.00345	0.04714
*GPATCH11*	0.000016	0.047
*MYOF*	0.02414	0.01266
*CXCL11*	0.000024	0.0048
*CCL8*	0.0016	0.0315
*ABTB2*	4.6E-06	0.017
*SMAD3*	0.0138	0.0165
*ABLIM3*	0.0028	0.0206
*CD9*	0.00022	0.00789
*S100A8*	0.02808	0.00012
*DUSP5*	0.0241	0.0043
*ALYREF*	0.00304	0.02158
*IDO1*	0.0023	0.0357
*MT1X*	0.044	0.031
*DEFB1*	0.0351	0.00026
*SOCS1*	0.01	0.025
*ANXA2R*	0.037	0.024

Furthermore, we used CIBERSORT to estimate the proportion of 22 immune cell types in bulk gene expression in respiratory samples from the RSV group, non-RSV group, and control group. In patients with RSV infection, we found that the proportion of immune cells from the innate immune system, including activated dendritic cells (DCs) and neutrophils, increased, while CD8-positive (CD8^+^) T cells and follicular helper T cells in the adaptive immune system significantly decreased compared with the non-RSV group and control group (Fig. 2B and Fig. S3). These results were in line with previous reports demonstrating increased DC and neutrophil counts and decreased T-cell counts in patients with RSV infection (11–13).

### Microbial diversity of the active microbiome among the RSV group, non-RSV group, and control group.

**FIG 5:**
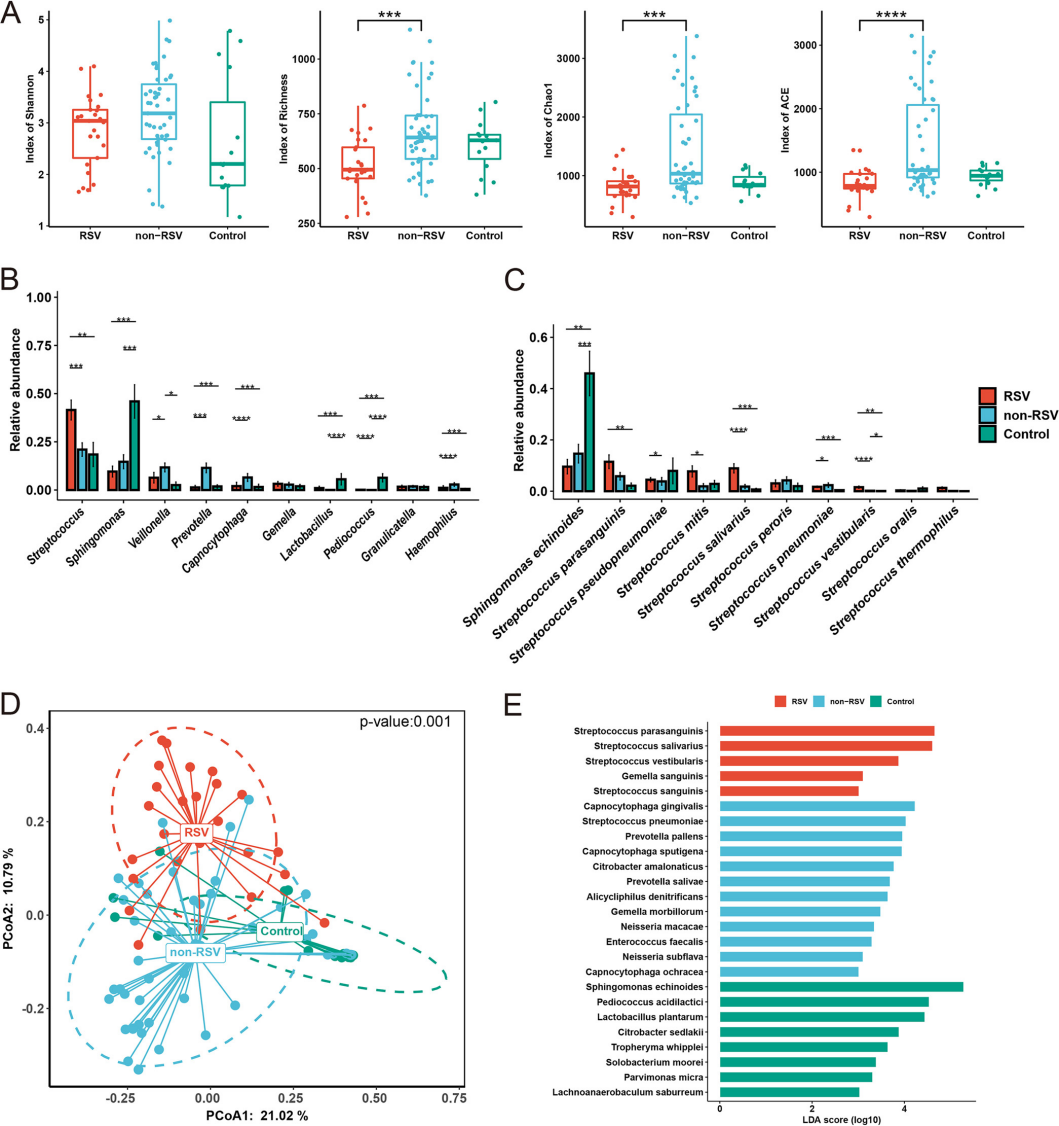
(A to E) Microbial community diversity within the RSV, non-RSV, and control groups. (A) Alpha diversity of the lung microbiota in cases of RSV, non-RSV, and control, measured by Shannon diversity index, richness index, Chao1 index, and ACE index. (B and C) Relative abundance of the top 10 most significantly enriched genera (B) and species (C) in the RSV, non-RSV, and control groups. (D) Composition of the upper respiratory microbiota in the RSV, non-RSV, and control groups, measured by principal-coordinate analysis via Jensen-Shannon divergence (JSD). (E) Specific species in the RSV, non-RSV, and control groups using linear discriminant analysis (LDA) score of >3; *P* = 0.05.

Next, we characterized the microbiome diversity from collected respiratory samples obtained by RNA-seq. According to our data, the Shannon diversity was not significantly different between the RSV group and non-RSV group (*P* > 0.05) or between the RSV group and control group (*P* > 0.05) (Fig. 5A). The non-RSV group had a significantly greater richness index (richness, Chao1, and ACE) than the RSV group (*P* < 0.001) (Fig. 5A), and the RSV group exhibited a relatively low richness among these three groups (Fig. 5A). Streptococcus was considerably more prevalent in the RSV group than in the non-RSV group or control group at the level of the top 10 genera (median, 41.5%; false-discovery rate [*q*] = 0.05) (Fig. 5B). *Prevotella* was, however, enriched in the non-RSV group (median, 11.5%; *q* = 0.05) (Fig. 5B). These two genera occupy the oral cavity and upper respiratory tract. Among Streptococcus genera, Streptococcus parasanguinis exhibited considerably higher abundance in the RSV group than in the control group, while Streptococcus salivarius in the RSV group had the highest abundance among the three groups (Fig. 5C). The total microbial composition (β-diversity) analysis revealed significant differences among the RSV group, non-RSV group, and control group (*P* = 0.001; permutational multivariate analysis of variance [PERMANOVA] test) (Fig. 5D). Then, we utilized linear discriminant analysis (LDA) to investigate specific species within distinct groups. Streptococcus parasanguinis, Streptococcus salivarius, Streptococcus vestibularis, and Gemella sanguinis were significantly enriched in the RSV group, whereas Capnocytophaga gingivalis, Capnocytophaga sputigena, and the other 10 species were more prevalent in the non-RSV group (LDA score > 3, *P* = 0.05) (Fig. 5E). To validated prevalence of Streptococcus both in the RSV and non-RSV groups, we did 16S rRNA sequencing to confirm the relative abundance of Streptococcus of metatranscriptome. We found a significant correlation of relative abundance between the two methods (*R* = 0.7, *P* = 3.3E-09) (Fig. S4A and B).

### Interaction between the active lung microbiome and host gene expression.

**FIG 6:**
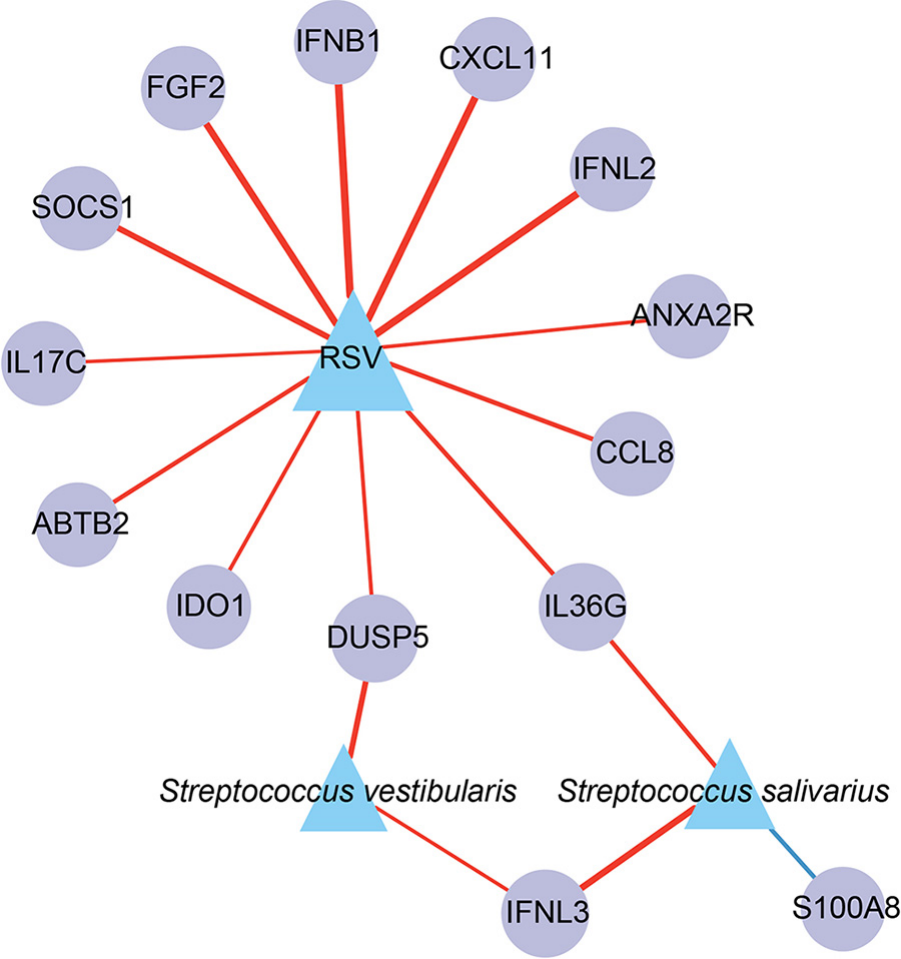
Network inference through co-occurrence analysis reveals gene-microbe associations of the immune-related and interferon-stimulated genes. Gray lines denote interactions between nodes (representing both microbes and host genes), with line thickness reflecting their observed interaction strength. Microbes within the co-occurrence network are color coded with respect to their groups.

To evaluate the relationship between the microbiome in the upper respiratory tract and systemic host responses induced by diverse viral infections, we examined host genes and species that were differentially expressed and active in the three groups. We eliminated correlation scores above 0.3 and *P*_adj_ values below 0.05. The results revealed strong interactions (including correlation) between IRGs, ISGs, respiratory syncytial viruses, Streptococcus salivarius, and Streptococcus vestibularis (the top two species of airway microbiota enriched in RSV samples) (Fig. 6), enabling the inference of gene-microbe connections within an integrated network. We concentrated on the differential IRGs and ISGs since they are a crucial component of the core host signature and are a group of genes that respond to RSV infections. RSV was strongly linked to numerous IRGs and ISGs, such as *CXCL11*, *SOCS1*, *IFNB1*, *IFNL2*, *DEFB1*, and *FGF2* (Fig. 6). Intriguingly, Streptococcus salivarius and Streptococcus vestibularis, two species significantly upregulated in the RSV group, were found to interact with RSV indirectly via *IL36G* and *DUSP5*, respectively, rather than directly (Fig. 6). Since *IL36G* and *DUSP5* showed significant increases in our transcriptome analysis, we further tested the results via qPCR. Transcription and secretion levels of *IL36G* were significantly upregulated in RSV infection *in vitro* samples (Fig. 3 and 4). This implied that RSV may alter the microenvironment of the upper respiratory tract by altering the expression level of immune genes in patients, or otherwise, the presence of specific microbes colonizing the upper respiratory tract might affect the expression of specific immune genes to favor RSV infection.

## DISCUSSION

In this article, we identified 2,063 RSV-specific gene signature DEGs in the RSV group compared with the non-RSV and control groups. Most of these RSV-specific DEGs exhibited downregulating patterns. While these genes were subjected to GO, KEGG, and Reactome pathway enrichment analysis, the majority of them were enriched in cilium function-related pathways. The mucociliary clearance system is the first line of defense of the respiratory tract, which initially removes pathogens through the mechanical mucus secretion of the respiratory epithelium and the pulsation of motor cilia (14–16). RSV preferentially infects airway ciliated epithelial cells (17), causing loss of cilium, overproduction of mucus, and necrosis of ciliated cells. Bronchiolitis is frequently associated with RSV infections and is characterized by mucus plug formation in the bronchiole lumens, partially due to loss of function of the mucociliary clearance system (18). RSV infection promotes significant ciliary dyskinesia in the early phase (19), and a sharp decrease in the ciliary beat frequency a few hours post-RSV infection has been reported (20). Ultrastructural studies revealed abnormalities in RSV-infected cells, characterized by nondirectional tubular systems and atypical axonemal basal bodies (21). The expression of β-tubulin and two ciliogenesis-related genes, *FOXJ1* and *DNAI2*, also decreased (21). Overall, RSV infection could lead to functional changes in the cilium and impair cilium organization and assembly. In addition to cilium-related pathways, most genes were enriched in extracellular structure organization and extracellular matrix organization pathways in GO BP enrichment analysis. According to GO CC and GO MF enrichment analysis, most of the enriched genes also referred to extracellular matrix components and extracellular matrix structural constituents. Interestingly, enrichment of extracellular matrix-related genes was specific to RSV infection compared with both the non-RSV infection and control groups. Studies have indicated that RSV infection could potentiate changes in extracellular matrix dynamics, which might be linked to asthma development (22–24). RSV-infected human lung fibroblasts could induce hyaluronan-enriched extracellular matrix production, promoting the adhesion of mast cells, which have long been recognized as a type of critical cell in the development of allergic airway inflammation and asthma. The binding between mast cells and the ECM was accompanied by augmented expression of proteases, chymase, and tryptase by mast cells, which led to a proinflammatory milieu and tissue damage during RSV infections (22). An *in vivo* study on juvenile and adult RSV-infected mice suggested that sustained myeloid infiltration observed in juvenile mice was related to extracellular matrix remodeling, facilitating the conditions for the development of asthma later in life (25). Xu et al. indicated that RSV infection could change the topographical map of chromatin, forming nucleosome-free regions on genes responsible for ECM production and remodeling, including *TGFB1*, *MMP9*, and *GFPT2*, which encode the rate-limiting enzyme in the hexosamine biosynthetic pathway (26), leading to epigenetic reprogramming and tissue remodeling (26). Our study provided evidence at the transcript level that RSV infection could alter ECM-related gene expression patterns, leading to extracellular matrix production and airway remodeling, and might be associated with long-term airway hyperresponsiveness, which makes those infected with RSV in early life predisposed to asthma.

We also found that *CXCL11* and *IDO1* genes exhibited high expression levels in the RSV group. *CXCL11*, also known as interferon gamma (IFN-γ)-inducible protein 9 (IP-9) or interferon-inducible T-cell alpha chemoattractant (I-TAC), is an immune chemokine belonging to the CXC family of chemokines and mainly regulates immune cell recruitment, differentiation, migration, and activation (27). It is induced by IFN-γ and IFN-β and weakly by IFN-α and has the highest affinity for CXCR3 (28). Studies have indicated that CXCR3 signaling pathways play a crucial role in the priming and activation of CD4^+^ T cells and in the balance between effector CD4^+^ T cells (Th1 and Th17) and regulatory CD4^+^ T cells (Th2 and Tr1) (29). The CXCL11-CXCR3 axis has been linked to inflammation, autoimmune disease, and cancer. The binding of CXCL11 to CXCR3 leads to internalization of CXCR3 (30), making it less accessible to CXCL9/CXCL10 and shifting the Th1/Th2 balance into Th2 (29). CXCL11/CXCR3 binding induces an immunotolerizing state via p70 kinase/mTOR in STAT3- and STAT6-dependent pathways, characterized by high levels of interferon 10 (IL-10^hi^) Tr1 and IL-4^hi^ Th2 cells, to restrain inflammation (31). Many studies have indicated that CXCL11 levels increase to various extents in response to viral infections, such as influenza virus (32), SARS-CoV-2 (33), human immunodeficiency virus (34), and hepatitis B virus (35). During RSV infection, Th2-biased polarization, which might be a consequence of high levels of CXCL11, may be associated with severe clinical manifestations. Severe/hypoxic bronchiolitis exhibited a higher level of IL-4 (a Th2 marker) than that of a group with nonhypoxic bronchiolitis (36). A higher IL-4/IFN-γ ratio in nasal lavage fluid was detected in RSV-infected infants with acute bronchiolitis than in their counterparts with only RSV-related upper respiratory tract infection alone (37). A cohort study also indicated that children with hypoxic RSV low respiratory tract infection showed a predominance of Th2 cytokines (38). Thus, our results indicated that *CXCL11* might play an important role in the pathogenesis of RSV infection by inducing a Th2 polarization immune response, although further studies are needed to investigate the specific mechanism.

Indoleamine 2,3-dioxygenase (IDO) is a rate-limiting enzyme involved in tryptophan-related metabolites (39). IDO regulates the immune response *in vitro* and *in vivo*, mainly shifting the Th balance toward Th2 and in favor of the Th2-related cytokine profile (40). It was also indicated that IDO was involved in the selective apoptosis of proliferating Th1 cells (40). Only a few studies have focused on the relationship between RSV and IDO, and these results are debatable. A study indicated that IDO played a protective role during RSV infection. The antiviral ability of IFN-γ in RSV pathogenesis was probably mediated by IDO since IDO was potently induced by IFN-γ and IDO knockdown *in vitro* could diminish the antiviral effect of IFN-γ (41). However, other studies mainly focused on the adverse effects of IDO during RSV infection. The upregulation of IDO in RSV-infected mesenchymal stem cells might affect immune cell proliferation, which might be responsible for the lack of protective immunity against RSV and for the long-term outcomes of RSV-induced lung diseases such as asthma and chronic obstructive pulmonary disease (42). IDO bioactivity and expression could be induced by RSV in monocyte-derived DCs in a virus replication-dependent manner, which inhibited T-bet (a Th1-cell marker) expression on T cells and thus led to Th2 polarization. A lower Th1 response to RSV might hinder viral clearance and probably promote the development of allergic diseases (43). Jin et al. showed that RSV infection reduced kynurenic acid production and inhibited the transformation from Th17 to Foxp3^+^ regulatory T cells (Tregs) (Th17/Treg balance) by altering IDO activity in plasmacytoid DCs (pDCs) (44), which might disrupt asthma tolerance later in life (45). High expression of *IDO1* in bat neutrophils was observed in a single-cell transcriptome analysis study of viral infection response in bats, which may play an important role in limiting inflammation in the context of neutrophil-activated conditions (46). In addition, El-Zaatari, M. et al. showed that neutrophil recruitment to infected cecum was reduced in *IDO1*-deficient mice (47), while the increase of local concentration of l-kynurenine, a reaction product of *IDO1*, inhibited neutrophil entry into infected tissues (48). Whether RSV utilizes IDO1 to attenuate neutrophil chemotaxis in lung tissue and thus reduce lung immune response remains to be investigated.

Our results are in accordance with previous studies that indicated that neutrophils constitute the major part of infiltrating cells in the airways of infants with RSV bronchiolitis (11). Neutrophils exert antimicrobial effects by phagocytosis, degranulation, secretion of broad antimicrobial proteins, and formation of neutrophil extracellular traps (NETs) (49). Neutrophils can help eliminate infected cells and reduce the amount of infectious virus *in vitro* (50). Human cathelicidin LL-37, which is primarily expressed by neutrophils and epithelial cells, has antiviral activity against RSV by directly affecting RSV viral particles and exerts protective effects against cell death (51). RSV infection could induce classical reactive oxygen species (ROS)-dependent NET release, which was able to bind and isolate RSV virion particles, inhibiting them from reaching the target cells (52). Serine proteases released from NETs can also interact with and inactivate RSV (53). However, deteriorating effects such as tissue damage and systemic symptoms come with the influx and infiltration of neutrophils in response to RSV infections. Studies have revealed that the extent of neutrophil infiltration is positively associated with clinical severity (54, 55). Greater neutrophil transepithelial migration, activation, and degranulation were associated with poorer ciliary function and more severe epithelial cell damage (56). Infiltration of neutrophils accumulating in the small airway impairs gas exchange (57) and promotes mucus production, which also leads to airway obstruction (58). Activated neutrophils can release antimicrobial mediators, such as elastase (59) and neutrophil-derived ROS, exerting antiviral effects (60), but are also cytotoxic to host cells at the same time. Thus, although neutrophils could help us eliminate the infection, inappropriate activation of neutrophils during RSV infection would lead to adverse outcomes. In addition to neutrophils, we found that DCs in the RSV group were significantly higher than those in the non-RSV group and control group. DCs can initiate and regulate adaptive immune responses according to peripheral cues, forming a remarkable cellular network against foreign and self-antigens (61). Although DCs may not be optimal permissive cells for RSV, RSV infects DCs probably as a strategy to interfere with the activation of antigen-specific immune responses and assist their infectious process indirectly. The interaction between RSV-infected DCs and T cells could evade adaptive immunity and inhibit T-cell-mediated virus clearance by interfering with immunological synapse assembly (62). RSV-exposed monocyte-derived DCs upregulate IFN-α and IFN-γ, suppressing the proliferation of CD4^+^ T cells (63). RSV could also alter the balance in the proportion of DC phenotypes in pathogenesis. pDCs play a protective role during RSV, characterized by controlling RSV replication and enhancing their elimination, suppressing pulmonary inflammation, and inhibiting the development of airway hyperresponsiveness (64), while lung myeloid DCs (mDCs) may have detrimental effects on the host during RSV infection (13). The number of pDCs was lower and that of mDCs was higher in the peripheral blood of children with RSV bronchiolitis than in healthy controls, and they still exhibited significantly lower quantities of pDCs even 3 months after discharge (65). Allergen sensitization and severe lung pathology caused by RSV infection are likely responsible for the accumulation and activation of mDCs, which might skew Th cell polarization toward a pathogenic direction. The turbulence caused by RSV in DC balances might have a long-term effect on the host and be responsible for the development of asthma later in life (66).

The differences in microbial diversity and composition among the RSV group, non-RSV group, and control group were also analyzed. We demonstrated that patients with RSV infection exhibited a relatively lower microbiological richness in their respiratory tract than patients in the non-RSV group or control group. Compared with the other groups, the RSV group was associated with a change in the abundance of specific microbiota, distinguished by a significant increase in Streptococcus, which was in accordance with previous studies (7, 10). Among the species of Streptococcus, the abundance of Streptococcus salivarius and Streptococcus vestibularis was significantly higher than that in either the non-RSV or control groups, indicating that these two species of Streptococcus might be involved in the pathogenesis of RSV infection. Integrative analysis of the active lung microbiome and transcriptome data could help us gain insight into the relationship between microbial composition and host genes. Thus, using gene and microbe coexpression network analysis, we characterized associations between host responses to RSV infection and the microbiome. RSV was positively correlated with some IRGs and ISGs, including *CXCL11*, *IDO1*, *DUSP5*, and *IL36G*. Among these genes, *IL36G*/IL-36γ was positively associated with Streptococcus salivarius, while *DUSP5* was positively correlated with Streptococcus vestibularis. IL-36 belongs to the IL-1 family, comprising IL-36α, IL-36β, IL-36γ, and IL-36Ra. IL-36γ can be expressed by macrophages, monocytes, and bronchial epithelial cells under stimulation by proinflammatory cytokines, including IL-1, tumor necrosis factor (TNF), IL-17, Pseudomonas aeruginosa, and Toll-like receptor 3 (TLR3) ligands (67). IL-36 is a potent regulator of DCs, characterized by upregulation of markers of activated DCs and increases in IL-6 and IL-12 production (68). IL-36γ might play a role in the development of allergen-induced inflammation in the lung. A study indicated that IL-36γ was upregulated in the lungs of asthma-susceptible A/J mice compared to asthma-resistant C3H/HeJ mice (69). Another study demonstrated that IL-36γ promoted airway hyperresponsiveness, induced neutrophil influx, enhanced NF-κB activity, and increased chemokine production (70). In addition, *IL-36γ* was significantly upregulated at the mRNA level in RSV-infected A549 cells. Thus, *IL-36γ* might contribute to the pathogenesis of RSV infection and potentially favor secondary Streptococcus salivarius infection. The expression of *DUSP5* was upregulated in clinical samples and also elevated in *in vitro* samples. We presumed that *DUSP5* was indirectly involved in RSV infection in the presence of Streptococcus vestibularis, which was absent in cell cultures. DUSPs are key regulators in the MAPK pathway that dephosphorylate key signaling molecules (71). DUSP5 shows phosphatase activity toward ERK (one of the best-known members of the MAPK family), leading to both activation and nuclear translocation of ERK (72). ERK-1/2 activity is required for efficient RSV infection and replication (73). Additionally, RSV-induced ERK-1/2 expression was involved in the posttranscriptional upregulation of *RANTES*, which was linked to severe RSV disease (74, 75). Thus, *DUSP5* probably promotes RSV infection via the ERK pathway. However, our study only indicated that RSV might interact with Streptococcus salivarius and Streptococcus vestibularis via *IL36G* and *DUSP5*, respectively. If RSV infection leads to a change in the abundance of these two bacteria via these two genes, further studies will be needed to elucidate the specific mechanisms.

One limitation of the present work was the relatively small sample size due to the difficulty encountered in collecting respiratory specimens of children, thus restricting the statistical power of the analysis used for identifying differences between groups. As a result, we only concentrated on the characteristics that were most closely associated with upper respiratory virus, microbiota, and host immune response. This may have made it impossible for the inquiry to identify more than a small number of potential microbiome types. The true respiratory microbiomes may be more diverse, and they may have more complex interactions with the human immune system. Nevertheless, the information pertaining to the stratified organization of the host immune signature and its correlation with different traits is statistically significant and supports the notion of an ongoing host-microbe interaction. Based on previous studies on metagenome data, it is alluring to anticipate that the variance in the microbiome is stratified in several dimensions in distinct viral infections. This stratification may represent changes in the homeostasis of the host and microbiome caused by various viruses.

Collectively, the comparative transcriptomics study of respiratory samples sheds light on the significant roles that ciliary organization and assembly, extracellular matrix changes, and microbial interactions play in the pathogenesis of RSV infection. Additionally, it was demonstrated that the recruitment of neutrophils and DCs in the respiratory tract is more substantial in RSV infection than in other viral infections. Finally, we discovered that RSV infection dramatically increased the expression of two ISGs (*CXCL11* and *IDO1*) and the abundance of Streptococcus.

## MATERIALS AND METHODS

### Subjects and clinical samples.

Nasopharyngeal swabs (NPs) or sputum samples (SPs) were collected from children diagnosed with pneumonia in the National Multicenter Pneumonia Pathogen Surveillance Program organized by Beijing Children’s Hospital. Total white blood cell counts less than 12 × 10^9^/L, C-reactive protein concentration in serum less than 20 mg/L, procalcitonin concentration in serum less than 0.5 ng/mL, and negative sputum culture were considered to rule out bacterial infection on the first routine blood test after admission (76). Samples with only one respiratory virus pathogen identified by PCR analysis were included in the study. Other inclusion criteria were age of less than 18 years and the presence of a parent or guardian capable of providing informed consent. Exclusion criteria included any underlying medical condition that required regular medical care, receipt of immunosuppressive medications, including corticosteroids, within the preceding 30 days and receipt of antibiotics within 7 days. Children hospitalized for selected surgery (such as hypospadias or hydronephrosis) and with no signs of any infection were enrolled as a control group. Demographic and laboratory parameters were obtained for each enrolled patient (see Table S1 in the supplemental material).

### Metatranscriptome sequencing and data processing.

According to the manufacturer's recommendations, a 1-μL sample was processed with Turbo DNase (Life Technologies, USA) to deplete the host DNA background. RNA was extracted using a QIAamp UCP pathogen minikit (Qiagen, Valencia, CA, USA), reverse transcribed, and amplified using an Ovation RNA-Seq system (NuGen, CA, USA). Following fragmentation, the library was constructed using Ovation Ultralow system v2 (NuGen, CA, USA) and sequenced on an Illumina NextSeq 550 (single-end 75 bp) (77). Raw sequencing data were processed using fastp (78) to remove reads containing adapters or ambiguous “N” nucleotides and low-quality reads. All clean data were mapped to the human genome hg19 using HISAT2 (79) with default parameters. FeatureCounts (80) was used to quantify expression at the gene level. Read count normalization and differentially expressed analyses were performed using the DESeq2 package (81). Differentially expressed genes (DEGs) were determined with an adjusted *P* value of <0.05 and absolute log fold change (log_2_FC) of ≥2. The clusterProfiler package (82) was used for gene ontology (GO), Kyoto Encyclopedia of Genes and Genomes (KEGG), and Reactome pathway enrichment analysis of DEGs. Benjamini-Hochberg-adjusted *P* values of <0.05 showed significant enrichment. The immune-related gene (IRG) list was downloaded from the Immport database (83), and interferon (IFN)-stimulated genes (ISGs) were retrieved from the study of Wu et al. (84). According to the gene expression profile of IRGs and ISGs, the Kruskal-Wallis test was used to select genes that were significantly different from the other two groups in the RSV group and visualized by the pheatmap package (85). To infer the composition of immune cells, the CIBERSORT algorithm (86) with the original CIBERSORT gene signature file LM22 and 1,000 permutations were used to examine the relative proportions of 22 invasive immune cell types in each sample.

### Microbial taxonomy assignment and host-microbial network interaction.

Taxonomy assignment of the upper respiratory microbiome was performed by Kraken 2 (87) (https://ccb.jhu.edu/software/kraken2/) with the Standard database (https://benlangmead.github.io/aws-indices/k2). The taxonomic relative abundance was calculated using Bracken (version 2.5). The alpha diversity was calculated using the Shannon index, richness, and Chao1 and ACE estimators. Wilcoxon rank sum tests were used to evaluate the differences in α-diversity and species richness among the three different groups (77). For β-diversity analysis, the principal-coordinate analysis scaling (PCoA) plot was based on Jensen-Shannon divergence (JSD) (88). The PERMANOVA test was used to determine the overall community composition difference between the elderly and young samples. These calculations and visualizations were implemented using the R package vegan. To elucidate the microbial and host co-occurrence patterns, species with frequencies greater than 40% were kept for correlation calculations using the SparCC algorithm (89). Only significant (*P* < 0.05) and robust (SparCC |*r*| > 0.3) correlations were retained for further co-occurrence network analyses. Visualization of networks was then implemented in Cytoscape (90).

### 16S rRNA sequencing and data processing.

DNA was extracted using a QIAamp UCP pathogen minikit (Qiagen, Valencia, CA, USA). The V3-V4 hypervariable region of the bacterial 16S rRNA gene was amplified with barcoded primer set 341F (CCTAYGGGRBGCASCAG) and 806R (GGACTACNNGGGTATCTAAT) with an expected amplicon length of 450 to 475 bp. Sequencing of the amplicons was performed using an Illumina NovaSeq 6000 instrument (Illumina, USA) (250-bp read length, paired-end protocol). Reads were analyzed by QIIME2 using the SILVA database (91, 92).

### Cells and viruses.

Type II alveolar epithelial cells (A549) (CCL-185) were obtained from ATCC (Manassas, VA, USA). The cells were maintained in Dulbecco’s modified Eagle medium (DMEM; Gibco) supplemented with 10% fetal bovine serum (Gibco) and 1% penicillin-streptomycin solution (Gibco) at 37°C and 5% CO_2_. RSV strain A2, human adenovirus type 7 (HAdV-7), and influenza A strain PR8 were propagated in A549 cells before quantification of viral titer by 50% tissue culture infective dose (TCID_50_) assay.

### Cell infections, qPCR, and ELISA.

All *in vitro* infection experiments were performed with a multiplicity of infection (MOI) of 0.1. *In vitro* samples were collected at 24, 48, and 72 h postinfection, cells were subsequently lysed with RLT buffer, and RNA was extracted using a Qiagen RNeasy kit (Qiagen, Valencia, CA, USA). cDNA was reverse transcribed from RNA extracts (500 ng) using a PrimeScript one-step reverse transcriptase PCR (RT-PCR) kit version 2 (TaKaRa, Japan). qPCR was carried out using TB Green premix Ex Taq (Tli RNase H Plus) (TaKaRa, Japan) with the primers listed in Table 3. Fold changes of target genes in infected samples were calculated by using the threshold cycle (ΔΔ*C_T_*) method and normalized to a GAPDH (glyceraldehyde-3-phosphate dehydrogenase) mRNA level (93). Cell-free supernatants were frozen and thawed before quantification of CXCL11 and IL36G was performed using a human CXCL11 enzyme-linked immunosorbent assay (ELISA) kit (Abcam) and human IL36G ELISA kit (Signalway Antibody), respectively. Statistics were performed with GraphPad Prism 8 software using unpaired *t* test. *P* values of <0.05 (*), <0.01 (**), and <0.001 (***) were considered significant. Bars in the figure panels show means and standard errors of the means (SEMs).

**TABLE 3:** Primer sequences used in this study

Gene name	Direction	Sequence (5′–3′)
*CXCL11*	Forward	GACGCTGTCTTTGCATAGGC
	Reverse	GGATTTAGGCATCGTTGTCCTTT
*IDO1*	Forward	TCTCATTTCGTGATGGAGACTGC
	Reverse	GTGTCCCGTTCTTGCATTTGC
*IL36G*	Forward	AGGAAGGGCCGTCTATCAATC
	Reverse	CACTGTCACTTCGTGGAACTG
*DUSP5*	Forward	GCGACCCACCTACACTACAAA
	Reverse	CTTCATAAGGTAAGCCATGCAGA

### Ethics statement.

This study was performed in strict accordance with the human subject protection guidance and was approved by the Ethical Review Committee of Beijing Children’s Hospital. Written informed consent was obtained from the participants’ parents or guardians.

### Data availability.

Meta-transcriptomic sequencing data were deposited in the Genome Warehouse in the National Genomics Data Center (National Genomics Data Center Members and Partners, 2021) under project PRJCA014022, which is publicly accessible at https://ngdc.cncb.ac.cn/.

## References

[1] Shi T, McAllister DA, O'Brien KL, Simoes EAF, Madhi SA, Gessner BD, Polack FP, Balsells E, Acacio S, Aguayo C, Alassani I, Ali A, Antonio M, Awasthi S, Awori JO, Azziz-Baumgartner E, Baggett HC, Baillie VL, Balmaseda A, Barahona A, Basnet S, Bassat Q, Basualdo W, Bigogo G, Bont L, Breiman RF, Brooks WA, Broor S, Bruce N, Bruden D, Buchy P, Campbell S, Carosone-Link P, Chadha M, Chipeta J, Chou M, Clara W, Cohen C, de Cuellar E, Dang D-A, Dash-Yandag B, Deloria-Knoll M, Dherani M, Eap T, Ebruke BE, Echavarria M, de Freitas Lázaro Emediato CC, Fasce RA, Feikin DR, Feng L, et al. 2017. Global, regional, and national disease burden estimates of acute lower respiratory infections due to respiratory syncytial virus in young children in 2015: a systematic review and modelling study. Lancet 390:946–958. doi:10.1016/S0140-6736(17)30938-8. 28689664 PMC5592248

[2] Blanken MO, Rovers MM, Molenaar JM, Winkler-Seinstra PL, Meijer A, Kimpen JLL, Bont L, Dutch RSV Neonatal Network. 2013. Respiratory syncytial virus and recurrent wheeze in healthy preterm infants. The N Engl J Med 368:1791–1799. doi:10.1056/NEJMoa1211917. 23656644

[3] Barr R, Green CA, Sande CJ, Drysdale SB. 2019. Respiratory syncytial virus: diagnosis, prevention and management. Ther Adv Infect Dis 6:2049936119865798. doi:10.1177/2049936119865798. 31384456 PMC6664627

[4] Mejias A, Cohen S, Glowinski R, Ramilo O. 2021. Host transcriptional signatures as predictive markers of infection in children. Curr Opin Infect Dis 34:552–558. doi:10.1097/QCO.0000000000000750. 34232136 PMC8446306

[5] Henrickson SE, Manne S, Dolfi DV, Mansfield KD, Parkhouse K, Mistry RD, Alpern ER, Hensley SE, Sullivan KE, Coffin SE, Wherry EJ. 2018. Genomic circuitry underlying immunological response to pediatric acute respiratory infection. Cell Rep 22:411–426. doi:10.1016/j.celrep.2017.12.043. 29320737 PMC5796675

[6] McDonald JU, Kaforou M, Clare S, Hale C, Ivanova M, Huntley D, Dorner M, Wright VJ, Levin M, Martinon-Torres F, Herberg JA, Tregoning JS. 2016. A simple screening approach to prioritize genes for functional analysis identifies a role for interferon regulatory factor 7 in the control of respiratory syncytial virus disease. mSystems 1:e00051-16. doi:10.1128/mSystems.00051-16. 27822537 PMC5069771

[7] de Steenhuijsen Piters WA, Heinonen S, Hasrat R, Bunsow E, Smith B, Suarez-Arrabal MC, Chaussabel D, Cohen DM, Sanders EA, Ramilo O, Bogaert D, Mejias A. 2016. Nasopharyngeal microbiota, host transcriptome, and disease severity in children with respiratory syncytial virus infection. Am J Respir Crit Care Med 194:1104–1115. doi:10.1164/rccm.201602-0220OC. 27135599 PMC5114450

[8] Yu J, Peterson DR, Baran AM, Bhattacharya S, Wylie TN, Falsey AR, Mariani TJ, Storch GA. 2019. Host gene expression in nose and blood for the diagnosis of viral respiratory infection. J Infect Dis 219:1151–1161. doi:10.1093/infdis/jiy608. 30339221 PMC6420164

[9] Stewart CJ, Mansbach JM, Wong MC, Ajami NJ, Petrosino JF, Camargo CA, Hasegawa K. 2017. Associations of nasopharyngeal metabolome and microbiome with severity among infants with bronchiolitis. A multiomic analysis. Am J Respir Crit Care Med 196:882–891. doi:10.1164/rccm.201701-0071OC. 28530140 PMC5649976

[10] Sonawane AR, Tian L, Chu C-Y, Qiu X, Wang L, Holden-Wiltse J, Grier A, Gill SR, Caserta MT, Falsey AR, Topham DJ, Walsh EE, Mariani TJ, Weiss ST, Silverman EK, Glass K, Liu Y-Y. 2019. Microbiome-transcriptome interactions related to severity of respiratory syncytial virus infection. Sci Rep 9:13824. doi:10.1038/s41598-019-50217-w. 31554845 PMC6761288

[11] Everard ML, Swarbrick A, Wrightham M, McIntyre J, Dunkley C, James PD, Sewell HF, Milner AD. 1994. Analysis of cells obtained by bronchial lavage of infants with respiratory syncytial virus infection. Arch Dis Child 71:428–432. doi:10.1136/adc.71.5.428. 7826113 PMC1030058

[12] Gill MA, Long K, Kwon T, Muniz L, Mejias A, Connolly J, Roy L, Banchereau J, Ramilo O. 2008. Differential recruitment of dendritic cells and monocytes to respiratory mucosal sites in children with influenza virus or respiratory syncytial virus infection. J Infect Dis 198:1667–1676. doi:10.1086/593018. 18847373 PMC2696361

[13] Gill MA, Palucka AK, Barton T, Ghaffar F, Jafri H, Banchereau J, Ramilo O. 2005. Mobilization of plasmacytoid and myeloid dendritic cells to mucosal sites in children with respiratory syncytial virus and other viral respiratory infections. J Infect Dis 191:1105–1115. doi:10.1086/428589. 15747246

[14] Chilvers MA, Callaghan CO. 2000. Local mucociliary defence mechanisms. Paediatr Respir Rev 1:27–34. doi:10.1053/prrv.2000.0009. 16263440

[15] Mall MA. 2008. Role of cilia, mucus, and airway surface liquid in mucociliary dysfunction: lessons from mouse models. J Aerosol Med Pulm Drug Deliv 21:13–24. doi:10.1089/jamp.2007.0659. 18518828

[16] Munkholm M, Mortensen J. 2014. Mucociliary clearance: pathophysiological aspects. Clin Physiol Funct Imaging 34:171–177. doi:10.1111/cpf.12085. 24119105

[17] Zhang L, Peeples ME, Boucher RC, Collins PL, Pickles RJ. 2002. Respiratory syncytial virus infection of human airway epithelial cells is polarized, specific to ciliated cells, and without obvious cytopathology. J Virol 76:5654–5666. doi:10.1128/jvi.76.11.5654-5666.2002. 11991994 PMC137037

[18] Meissner HC. 2016. Viral bronchiolitis in children. N Engl J Med 374:62–72. doi:10.1056/NEJMra1413456. 26735994

[19] Smith CM, Kulkarni H, Radhakrishnan P, Rutman A, Bankart MJ, Williams G, Hirst RA, Easton AJ, Andrew PW, O'Callaghan C. 2014. Ciliary dyskinesia is an early feature of respiratory syncytial virus infection. Eur Respir J 43:485–496. doi:10.1183/09031936.00205312. 23520320

[20] Philippou S, Otto P, Reinhold P, Elschner M, Streckert HJ. 2000. Respiratory syncytial virus-induced chronic bronchiolitis in experimentally infected calves. Virchows Arch 436:617–621. doi:10.1007/s004280000197. 10917178

[21] Mata M, Sarrion I, Armengot M, Carda C, Martinez I, Melero JA, Cortijo J. 2012. Respiratory syncytial virus inhibits ciliagenesis in differentiated normal human bronchial epithelial cells: effectiveness of N-acetylcysteine. PLoS One 7:e48037. doi:10.1371/journal.pone.0048037. 23118923 PMC3485262

[22] Reeves SR, Barrow KA, Rich LM, White MP, Shubin NJ, Chan CK, Kang I, Ziegler SF, Piliponsky AM, Wight TN, Debley JS. 2019. Respiratory syncytial virus infection of human lung fibroblasts induces a hyaluronan-enriched extracellular matrix that binds mast cells and enhances expression of mast cell proteases. Front Immunol 10:3159. doi:10.3389/fimmu.2019.03159. 32047499 PMC6997473

[23] Zhao Y, Qiao D, Skibba M, Brasier AR. 2022. The IRE1alpha–XBP1s arm of the unfolded protein response activates N-glycosylation to remodel the subepithelial basement membrane in paramyxovirus infection. Int J Mol Sci 23:9000. doi:10.3390/ijms23169000. 36012265 PMC9408905

[24] Tourdot S, Mathie S, Hussell T, Edwards L, Wang H, Openshaw PJM, Schwarze J, Lloyd CM. 2008. Respiratory syncytial virus infection provokes airway remodelling in allergen-exposed mice in absence of prior allergen sensitization. Clin Exp Allergy 38:1016–1024. doi:10.1111/j.1365-2222.2008.02974.x. 18498543 PMC3385350

[25] Kellar GG, Reeves SR, Barrow KA, Debley JS, Wight TN, Ziegler SF. 2020. Juvenile, but not adult, mice display increased myeloid recruitment and extracellular matrix remodeling during respiratory syncytial virus infection. J Immunol 205:3050–3057. doi:10.4049/jimmunol.2000683. 33097575 PMC7747670

[26] Xu X, Qiao D, Mann M, Garofalo RP, Brasier AR. 2020. Respiratory syncytial virus infection induces chromatin remodeling to activate growth factor and extracellular matrix secretion pathways. Viruses 12:804. doi:10.3390/v12080804. 32722537 PMC7472097

[27] Tokunaga R, Zhang W, Naseem M, Puccini A, Berger MD, Soni S, McSkane M, Baba H, Lenz H-J. 2018. CXCL9, CXCL10, CXCL11/CXCR3 axis for immune activation-a target for novel cancer therapy. Cancer Treat Rev 63:40–47. doi:10.1016/j.ctrv.2017.11.007. 29207310 PMC5801162

[28] Cole KE, Strick CA, Paradis TJ, Ogborne KT, Loetscher M, Gladue RP, Lin W, Boyd JG, Moser B, Wood DE, Sahagan BG, Neote K. 1998. Interferon–inducible T cell alpha chemoattractant (I-TAC): a novel non-ELR CXC chemokine with potent activity on activated T cells through selective high affinity binding to CXCR3. J Exp Med 187:2009–2021. doi:10.1084/jem.187.12.2009. 9625760 PMC2212354

[29] Karin N, Wildbaum G, Thelen M. 2016. Biased signaling pathways via CXCR3 control the development and function of CD4+ T cell subsets. J Leukoc Biol 99:857–862. doi:10.1189/jlb.2MR0915-441R. 26657511

[30] Colvin RA, Campanella GS, Sun J, Luster AD. 2004. Intracellular domains of CXCR3 that mediate CXCL9, CXCL10, and CXCL11 function. J Biol Chem 279:30219–30227. doi:10.1074/jbc.M403595200. 15150261

[31] Zohar Y, Wildbaum G, Novak R, Salzman AL, Thelen M, Alon R, Barsheshet Y, Karp CL, Karin N. 2014. CXCL11-dependent induction of FOXP3-negative regulatory T cells suppresses autoimmune encephalomyelitis. J Clin Invest 124:2009–2022. doi:10.1172/JCI71951. 24713654 PMC4001543

[32] Dissanayake TK, Schäuble S, Mirhakkak MH, Wu W-L, Ng AC-K, Yip CCY, López AG, Wolf T, Yeung M-L, Chan K-H, Yuen K-Y, Panagiotou G, To KK-W. 2020. Comparative transcriptomic analysis of rhinovirus and influenza virus infection. Front Microbiol 11:1580. doi:10.3389/fmicb.2020.01580. 32849329 PMC7396524

[33] Xiong Y, Liu Y, Cao L, Wang D, Guo M, Jiang A, Guo D, Hu W, Yang J, Tang Z, Wu H, Lin Y, Zhang M, Zhang Q, Shi M, Liu Y, Zhou Y, Lan K, Chen Y. 2020. Transcriptomic characteristics of bronchoalveolar lavage fluid and peripheral blood mononuclear cells in COVID-19 patients. Emerg Microbes Infect 9:761–770. doi:10.1080/22221751.2020.1747363. 32228226 PMC7170362

[34] Yin X, Wang Z, Wu T, Ma M, Zhang Z, Chu Z, Hu Q, Ding H, Han X, Xu J, Shang H, Jiang Y. 2019. The combination of CXCL9, CXCL10 and CXCL11 levels during primary HIV infection predicts HIV disease progression. J Transl Med 17:417. doi:10.1186/s12967-019-02172-3. 31836011 PMC6909626

[35] Koshiol J, Argirion I, Liu Z, Kim Lam T, O'Brien TR, Yu K, McGlynn KA, Petrick JL, Pinto L, Chen C-J, Hildesheim A, Pfeiffer RM, Lee M-H, Yang H-I. 2021. Immunologic markers and risk of hepatocellular carcinoma in hepatitis B virus- and hepatitis C virus-infected individuals. Aliment Pharmacol Ther 54:833–842. doi:10.1111/apt.16524. 34286851 PMC12332984

[36] Garofalo RP, Patti J, Hintz KA, Hill V, Ogra PL, Welliver RC. 2001. Macrophage inflammatory protein-1alpha (not T helper type 2 cytokines) is associated with severe forms of respiratory syncytial virus bronchiolitis. J Infect Dis 184:393–399. doi:10.1086/322788. 11471095

[37] Legg JP, Hussain IR, Warner JA, Johnston SL, Warner JO. 2003. Type 1 and type 2 cytokine imbalance in acute respiratory syncytial virus bronchiolitis. Am J Respir Crit Care Med 168:633–639. doi:10.1164/rccm.200210-1148OC. 12773328

[38] Bermejo-Martin JF, Garcia-Arevalo MC, De Lejarazu RO, Ardura J, Eiros JM, Alonso A, Matías V, Pino M, Bernardo D, Arranz E, Blanco-Quiros A. 2007. Predominance of Th2 cytokines, CXC chemokines and innate immunity mediators at the mucosal level during severe respiratory syncytial virus infection in children. Eur Cytokine Netw 18:162–167. doi:10.1684/ecn.2007.0096. 17823085

[39] Cole JE, Astola N, Cribbs AP, Goddard ME, Park I, Green P, Davies AH, Williams RO, Feldmann M, Monaco C. 2015. Indoleamine 2,3-dioxygenase-1 is protective in atherosclerosis and its metabolites provide new opportunities for drug development. Proc Natl Acad Sci USA 112:13033–13038. doi:10.1073/pnas.1517820112. 26438837 PMC4620898

[40] Fallarino F, Grohmann U, Vacca C, Bianchi R, Orabona C, Spreca A, Fioretti MC, Puccetti P. 2002. T cell apoptosis by tryptophan catabolism. Cell Death Differ 9:1069–1077. doi:10.1038/sj.cdd.4401073. 12232795

[41] Rajan D, Chinnadurai R, O'Keefe EL, Boyoglu-Barnum S, Todd SO, Hartert TV, Galipeau J, Anderson LJ. 2017. Protective role of indoleamine 2,3 dioxygenase in respiratory syncytial virus associated immune response in airway epithelial cells. Virology 512:144–150. doi:10.1016/j.virol.2017.09.007. 28963880 PMC5653408

[42] Cheung MB, Sampayo-Escobar V, Green R, Moore ML, Mohapatra S, Mohapatra SS. 2016. Respiratory syncytial virus-infected mesenchymal stem cells regulate immunity via interferon beta and indoleamine-2,3-dioxygenase. PLoS One 11:e0163709. doi:10.1371/journal.pone.0163709. 27695127 PMC5047639

[43] Ajamian F, Wu Y, Ebeling C, Ilarraza R, Odemuyiwa SO, Moqbel R, Adamko DJ. 2015. Respiratory syncytial virus induces indoleamine 2,3-dioxygenase activity: a potential novel role in the development of allergic disease. Clin Exp Allergy 45:644–659. doi:10.1111/cea.12498. 25627660

[44] Jin L, Hu Q, Hu Y, Chen Z, Liao W. 2020. Respiratory syncytial virus infection reduces kynurenic acid production and reverses Th17/Treg balance by modulating indoleamine 2,3-dioxygenase (IDO) molecules in plasmacytoid dendritic cells. Med Sci Monit 26:e926763. doi:10.12659/MSM.926763. 33262321 PMC7720431

[45] Shi T, Li N, He Y, Feng J, Mei Z, Du Y, Jie Z. 2021. Th17/Treg cell imbalance plays an important role in respiratory syncytial virus infection compromising asthma tolerance in mice. Microb Pathog 156:104867. doi:10.1016/j.micpath.2021.104867. 33957244

[46] Gamage AM, Chan WOY, Zhu F, Lim YT, Long S, Ahn M, Tan CW, Hiang Foo RJ, Sia WR, Lim XF, He H, Zhai W, Anderson DE, Sobota RM, Dutertre C-A, Wang L-F. 2022. Single-cell transcriptome analysis of the in vivo response to viral infection in the cave nectar bat Eonycteris spelaea. Immunity 55:2187–2205.e2185. doi:10.1016/j.immuni.2022.10.008. 36351376

[47] El-Zaatari M, Chang Y-M, Zhang M, Franz M, Shreiner A, McDermott AJ, van der Sluijs KF, Lutter R, Grasberger H, Kamada N, Young VB, Huffnagle GB, Kao JY. 2014. Tryptophan catabolism restricts IFN-γ-expressing neutrophils and Clostridium difficile immunopathology. J Immunol 193:807–816. doi:10.4049/jimmunol.1302913. 24935925 PMC4091639

[48] Loughman JA, Yarbrough ML, Tiemann KM, Hunstad DA. 2016. Local generation of kynurenines mediates inhibition of neutrophil chemotaxis by uropathogenic Escherichia coli. Infect Immun 84:1176–1183. doi:10.1128/IAI.01202-15. 26857571 PMC4807489

[49] Amulic B, Cazalet C, Hayes GL, Metzler KD, Zychlinsky A. 2012. Neutrophil function: from mechanisms to disease. Annu Rev Immunol 30:459–489. doi:10.1146/annurev-immunol-020711-074942. 22224774

[50] Deng Y, Herbert JA, Robinson E, Ren L, Smyth RL, Smith CM. 2020. Neutrophil-airway epithelial interactions result in increased epithelial damage and viral clearance during respiratory syncytial virus infection. J Virol 94:e02161-19. doi:10.1128/JVI.02161-19. 32295918 PMC7307165

[51] Currie SM, Findlay EG, McHugh BJ, Mackellar A, Man T, Macmillan D, Wang H, Fitch PM, Schwarze J, Davidson DJ. 2013. The human cathelicidin LL-37 has antiviral activity against respiratory syncytial virus. PLoS One 8:e73659. doi:10.1371/journal.pone.0073659. 24023689 PMC3758310

[52] Cortjens B, de Boer OJ, de Jong R, Antonis AF, Sabogal Piñeros YS, Lutter R, van Woensel JB, Bem RA. 2016. Neutrophil extracellular traps cause airway obstruction during respiratory syncytial virus disease. J Pathol 238:401–411. doi:10.1002/path.4660. 26468056

[53] Lopes BRP, da Silva GS, de Lima Menezes G, de Oliveira J, Watanabe ASA, Porto BN, da Silva RA, Toledo KA. 2022. Serine proteases in neutrophil extracellular traps exhibit anti-respiratory syncytial virus activity. Int Immunopharmacol 106:108573. doi:10.1016/j.intimp.2022.108573. 35183035

[54] Johnson JE, Gonzales RA, Olson SJ, Wright PF, Graham BS. 2007. The histopathology of fatal untreated human respiratory syncytial virus infection. Mod Pathol 20:108–119. doi:10.1038/modpathol.3800725. 17143259

[55] Lukens MV, van de Pol AC, Coenjaerts FEJ, Jansen NJG, Kamp VM, Kimpen JLL, Rossen JWA, Ulfman LH, Tacke CEA, Viveen MC, Koenderman L, Wolfs TFW, van Bleek GM. 2010. A systemic neutrophil response precedes robust CD8(+) T-cell activation during natural respiratory syncytial virus infection in infants. J Virol 84:2374–2383. doi:10.1128/JVI.01807-09. 20015982 PMC2820924

[56] Herbert JA, Deng Y, Hardelid P, Robinson E, Ren L, Moulding D, Smyth RL, Smith CM. 2020. Beta2-integrin LFA1 mediates airway damage following neutrophil transepithelial migration during respiratory syncytial virus infection. Eur Respir J 56:1902216. doi:10.1183/13993003.02216-2019. 32217648 PMC7406857

[57] Sande CJ, Njunge JM, Mwongeli Ngoi J, Mutunga MN, Chege T, Gicheru ET, Gardiner EM, Gwela A, Green CA, Drysdale SB, Berkley JA, Nokes DJ, Pollard AJ. 2019. Airway response to respiratory syncytial virus has incidental antibacterial effects. Nat Commun 10:2218. doi:10.1038/s41467-019-10222-z. 31101811 PMC6525170

[58] Stokes KL, Currier MG, Sakamoto K, Lee S, Collins PL, Plemper RK, Moore ML. 2013. The respiratory syncytial virus fusion protein and neutrophils mediate the airway mucin response to pathogenic respiratory syncytial virus infection. J Virol 87:10070–10082. doi:10.1128/JVI.01347-13. 23843644 PMC3753991

[59] Cavarra E, Martorana PA, Gambelli F, de Santi M, van Even P, Lungarella G. 1996. Neutrophil recruitment into the lungs is associated with increased lung elastase burden, decreased lung elastin, and emphysema in alpha 1 proteinase inhibitor-deficient mice. Lab Invest 75:273–280. 8765327

[60] Hosakote YM, Jantzi PD, Esham DL, Spratt H, Kurosky A, Casola A, Garofalo RP. 2011. Viral-mediated inhibition of antioxidant enzymes contributes to the pathogenesis of severe respiratory syncytial virus bronchiolitis. Am J Respir Crit Care Med 183:1550–1560. doi:10.1164/rccm.201010-1755OC. 21471094 PMC3137144

[61] Merad M, Sathe P, Helft J, Miller J, Mortha A. 2013. The dendritic cell lineage: ontogeny and function of dendritic cells and their subsets in the steady state and the inflamed setting. Annu Rev Immunol 31:563–604. doi:10.1146/annurev-immunol-020711-074950. 23516985 PMC3853342

[62] González PA, Prado CE, Leiva ED, Carreño LJ, Bueno SM, Riedel CA, Kalergis AM. 2008. Respiratory syncytial virus impairs T cell activation by preventing synapse assembly with dendritic cells. Proc Natl Acad Sci USA 105:14999–15004. doi:10.1073/pnas.0802555105. 18818306 PMC2567482

[63] Chi B, Dickensheets HL, Spann KM, Alston MA, Luongo C, Dumoutier L, Huang J, Renauld J-C, Kotenko SV, Roederer M, Beeler JA, Donnelly RP, Collins PL, Rabin RL. 2006. Alpha and lambda interferon together mediate suppression of CD4 T cells induced by respiratory syncytial virus. J Virol 80:5032–5040. doi:10.1128/JVI.80.10.5032-5040.2006. 16641294 PMC1472058

[64] Wang H, Peters N, Schwarze J. 2006. Plasmacytoid dendritic cells limit viral replication, pulmonary inflammation, and airway hyperresponsiveness in respiratory syncytial virus infection. J Immunol 177:6263–6270. doi:10.4049/jimmunol.177.9.6263. 17056556

[65] Weng K, Zhang J, Mei X, Wu A, Zhang B, Cai M, Zheng Y, Ke Z. 2014. Lower number of plasmacytoid dendritic cells in peripheral blood of children with bronchiolitis following respiratory syncytial virus infection. Influenza Other Respir Viruses 8:469–473. doi:10.1111/irv.12242. 24528606 PMC4181807

[66] Silver E, Yin-DeClue H, Schechtman KB, Grayson MH, Bacharier LB, Castro M. 2009. Lower levels of plasmacytoid dendritic cells in peripheral blood are associated with a diagnosis of asthma 6 yr after severe respiratory syncytial virus bronchiolitis. Pediatr Allergy Immunol 20:471–476. doi:10.1111/j.1399-3038.2008.00818.x. 19140903 PMC3515331

[67] Chustz RT, Nagarkar DR, Poposki JA, Favoreto S, Avila PC, Schleimer RP, Kato A. 2011. Regulation and function of the IL-1 family cytokine IL-1F9 in human bronchial epithelial cells. Am J Respir Cell Mol Biol 45:145–153. doi:10.1165/rcmb.2010-0075OC. 20870894 PMC3145067

[68] Vigne S, Palmer G, Lamacchia C, Martin P, Talabot-Ayer D, Rodriguez E, Ronchi F, Sallusto F, Dinh H, Sims JE, Gabay C. 2011. IL-36R ligands are potent regulators of dendritic and T cells. Blood 118:5813–5823. doi:10.1182/blood-2011-05-356873. 21860022

[69] Ramadas RA, Li X, Shubitowski DM, Samineni S, Wills-Karp M, Ewart SL. 2006. IL-1 Receptor antagonist as a positional candidate gene in a murine model of allergic asthma. Immunogenetics 58:851–855. doi:10.1007/s00251-006-0146-x. 17021861

[70] Ramadas RA, Ewart SL, Medoff BD, LeVine AM. 2011. Interleukin-1 family member 9 stimulates chemokine production and neutrophil influx in mouse lungs. Am J Respir Cell Mol Biol 44:134–145. doi:10.1165/rcmb.2009-0315OC. 20299540 PMC3049228

[71] Caunt CJ, Keyse SM. 2013. Dual-specificity MAP kinase phosphatases (MKPs): shaping the outcome of MAP kinase signalling. FEBS J 280:489–504. doi:10.1111/j.1742-4658.2012.08716.x. 22812510 PMC3594966

[72] Mandl M, Slack DN, Keyse SM. 2005. Specific inactivation and nuclear anchoring of extracellular signal-regulated kinase 2 by the inducible dual-specificity protein phosphatase DUSP5. Mol Cell Biol 25:1830–1845. doi:10.1128/MCB.25.5.1830-1845.2005. 15713638 PMC549372

[73] Kong X, San Juan H, Behera A, Peeples ME, Wu J, Lockey RF, Mohapatra SS. 2004. ERK-1/2 activity is required for efficient RSV infection. FEBS Lett 559:33–38. doi:10.1016/S0014-5793(04)00002-X. 14960303

[74] Amanatidou V, Sourvinos G, Apostolakis S, Neonaki P, Tsilimigaki A, Krambovitis E, Spandidos DA. 2008. RANTES promoter gene polymorphisms and susceptibility to severe respiratory syncytial virus-induced bronchiolitis. Pediatr Infect Dis J 27:38–42. doi:10.1097/INF.0b013e31814d4e42. 18162936

[75] Pazdrak K, Olszewska-Pazdrak B, Liu T, Takizawa R, Brasier AR, Garofalo RP, Casola A. 2002. MAPK activation is involved in posttranscriptional regulation of RSV-induced RANTES gene expression. Am J Physiol Lung Cell Mol Physiol 283:L364–372. doi:10.1152/ajplung.00331.2001. 12114198

[76] Ruuskanen O, Lahti E, Jennings LC, Murdoch DR. 2011. Viral pneumonia. Lancet 377:1264–1275. doi:10.1016/S0140-6736(10)61459-6. 21435708 PMC7138033

[77] Ren L, Zhang R, Rao J, Xiao Y, Zhang Z, Yang B, Cao D, Zhong H, Ning P, Shang Y, Li M, Gao Z, Wang J. 2018. Transcriptionally active lung microbiome and its association with bacterial biomass and host inflammatory status. mSystems 3:e00199-18. doi:10.1128/mSystems.00199-18. 30417108 PMC6208642

[78] Chen S, Zhou Y, Chen Y, Gu J. 2018. fastp: an ultra-fast all-in-one FASTQ preprocessor. Bioinformatics 34:i884–i890. doi:10.1093/bioinformatics/bty560. 30423086 PMC6129281

[79] Kim D, Paggi JM, Park C, Bennett C, Salzberg SL. 2019. Graph-based genome alignment and genotyping with HISAT2 and HISAT-genotype. Nat Biotechnol 37:907–915. doi:10.1038/s41587-019-0201-4. 31375807 PMC7605509

[80] Liao Y, Smyth GK, Shi W. 2014. featureCounts: an efficient general purpose program for assigning sequence reads to genomic features. Bioinformatics 30:923–930. doi:10.1093/bioinformatics/btt656. 24227677

[81] Love MI, Huber W, Anders S. 2014. Moderated estimation of fold change and dispersion for RNA-seq data with DESeq2. Genome Biol 15:550. doi:10.1186/s13059-014-0550-8. 25516281 PMC4302049

[82] Guangchuang Y, Li-Gen W, Yanyan H, Qing-Yu H. 2012. clusterProfiler: an R package for comparing biological themes among gene clusters. Omics 16:284–287. doi:10.1089/omi.2011.0118. 22455463 PMC3339379

[83] Bhattacharya S, Andorf S, Gomes L, Dunn P, Schaefer H, Pontius J, Berger P, Desborough V, Smith T, Campbell J, Thomson E, Monteiro R, Guimaraes P, Walters B, Wiser J, Butte AJ. 2014. ImmPort: disseminating data to the public for the future of immunology. Immunol Res 58:234–239. doi:10.1007/s12026-014-8516-1. 24791905

[84] Wu X, Dao Thi VL, Huang Y, Billerbeck E, Saha D, Hoffmann H-H, Wang Y, Silva LAV, Sarbanes S, Sun T, Andrus L, Yu Y, Quirk C, Li M, MacDonald MR, Schneider WM, An X, Rosenberg BR, Rice CM. 2018. Intrinsic immunity shapes viral resistance of stem cells. Cell 172:423–438.e425. doi:10.1016/j.cell.2017.11.018. 29249360 PMC5786493

[85] Nam HH, Ison MG. 2019. Respiratory syncytial virus infection in adults. BMJ 366:l5021. doi:10.1136/bmj.l5021. 31506273

[86] Newman AM, Liu CL, Green MR, Gentles AJ, Feng W, Xu Y, Hoang CD, Diehn M, Alizadeh AA. 2015. Robust enumeration of cell subsets from tissue expression profiles. Nat Methods 12:453–457. doi:10.1038/nmeth.3337. 25822800 PMC4739640

[87] Wood DE, Lu J, Langmead B. 2019. Improved metagenomic analysis with Kraken 2. Genome Biol 20:257. doi:10.1186/s13059-019-1891-0. 31779668 PMC6883579

[88] Arumugam M, Raes J, Pelletier E, Le Paslier D, Yamada T, Mende DR, Fernandes GR, Tap J, Bruls T, Batto J-M, Bertalan M, Borruel N, Casellas F, Fernandez L, Gautier L, Hansen T, Hattori M, Hayashi T, Kleerebezem M, Kurokawa K, Leclerc M, Levenez F, Manichanh C, Nielsen HB, Nielsen T, Pons N, Poulain J, Qin J, Sicheritz-Ponten T, Tims S, Torrents D, Ugarte E, Zoetendal EG, Wang J, Guarner F, Pedersen O, de Vos WM, Brunak S, Doré J, Antolín M, Artiguenave F, Blottiere HM, Almeida M, Brechot C, Cara C, Chervaux C, Cultrone A, Delorme C, Denariaz G, Dervyn R, et al. 2011. Enterotypes of the human gut microbiome. Nature 473:174–180. doi:10.1038/nature09944. 21508958 PMC3728647

[89] Friedman J, Alm EJ. 2012. Inferring correlation networks from genomic survey data. PLoS Comput Biol 8:e1002687. doi:10.1371/journal.pcbi.1002687. 23028285 PMC3447976

[90] Treister A, Pico AR. 2018. Identifier mapping in Cytoscape. F1000Res 7:725. doi:10.12688/f1000research.14807.2. 30079244 PMC6053696

[91] Hall M, Beiko RG. 2018. 16S rRNA gene analysis with QIIME2. Methods Mol Biol 1849:113–129. doi:10.1007/978-1-4939-8728-3_8. 30298251

[92] Quast C, Pruesse E, Yilmaz P, Gerken J, Schweer T, Yarza P, Peplies J, Glöckner FO. 2013. The SILVA ribosomal RNA gene database project: improved data processing and web-based tools. Nucleic Acids Res 41:D590–D596. doi:10.1093/nar/gks1219. 23193283 PMC3531112

[93] Livak KJ, Schmittgen TD. 2001. Analysis of relative gene expression data using real-time quantitative PCR and the 2(-delta delta C(T)) method. Methods 25:402–408. doi:10.1006/meth.2001.1262. 11846609

