# Supplementary material for: Metatranscriptome Reveals Specific Immune and Microbial Signatures of Respiratory Syncytial Virus Infection in Children
Source: Microbiol Spectr. 2023 Mar 2;11(2):e04107-22. doi: 10.1128/spectrum.04107-22 (PMC10100699; doi:10.1128/spectrum.04107-22)
Supplement: Supplemental file 1 — Supplemental material. Download spectrum.04107-22-s0001.pdf, PDF file, 0.9 MB [file spectrum.04107-22-s0001.pdf]

Table S1. Demographic and laboratory parameters in in control, RSV and non-RSV groups.

	Control (n=15)	RSV (n=25)	non-RSV (n=53)		
			Flu	AdV	hMPV
**Age (months)**	37.00 [21.50, 55.25]	3.67 [2.07, 6.50]	11.00 [4.17, 35.00]	20.00 [10.65, 47.50]	10.00 [4.10, 18.50]
**Gender**					
Male	9 (60%)	15 (60%)	16 (84%)	8 (62%)	14 (67%)
Female	6 (40%)	10 (40%)	3 (16%)	5 (38%)	7 (33%)
**Baseline laboratory values**					
White blood cells/mm3	7.76 [6.61, 8.57]	8.70 [7.37, 10.34]	8.60 [6.30, 9.60]	7.50 [5.74, 9.65]	9.20 [5.60, 10.83]
Neutrophil percent	39.55 [29.65, 45.28]	20.60 [14.25, 29.55]	32.00 [21.11, 38.45]	29.50 [24.95, 43.90]	36.00 [30.40, 41.30]
Lymphocyte percent	52.15 [47.08, 60.40]	65.80 [59.60, 73.20]	57.30 [52.04, 70.26]	57.10 [45.40, 64.05]	52.40 [49.00, 60.20]
C-reactive protein (mg/L)	N/A	1.50 [0.51, 3.85]	1.50 [0.50, 4.00]	1.45 [0.79, 14.05]	5.00 [1.09, 9.47]
Procalcitonin (ng/mL)	N/A	0.12 [0.09, 0.31]	0.10 [0.09, 0.12]	0.13 [0.05, 0.28]	0.10 [0.05, 0.14]

Data reported as median [IQR] or number (percent).

N/A = not available

Figure S1. Principal Coordinate Analysis of RNA expression between 25 sputum samples (SPs) and 53 nasopharyngeal swabs (NPs) (PERMANOVA p-values = 0.001, without multiple comparisons, respectively).

Figure S2. Detailed expression levels of immune-related genes (IRGs) and interferon-stimulated genes (ISGs) which were screened by P value < 0.05 in RSV group compared to control and non-RSV group respectively.

Figure S3. CIBERSORT was used to analyze the proportion of 22 immune cell types in bulk gene expression in respiratory samples from RSV, non-RSV and control groups. Among them, the proportion of 15 immune cell types were found to be significantly different in RSV group as compared to both non-RSV and control groups.

Figure S4. 16s rRNA sequencing was performed to confirm relative abundance of *Streptococcus* of metatranscriptome. (A) Correlation of Streptococcus between 16s rRNA sequencing and metatranscritpome. X axis represents relative abundance of *Streptococcus* in 16s rRNA sequencing data and Y axis represents relative abundance of *Streptococcus* in metatranscritpome sequencing data. Each point represents one sample. (B) Relative abundance of bacterial communities at the genus level for each respiratory sample (n = 1 per patient) from 53 patients, identified via bulk 16S rRNA gene amplicon sequencing. Respiratory specimens (n = 53) are denoted as RSV group and non-RSV group.

Figure S1

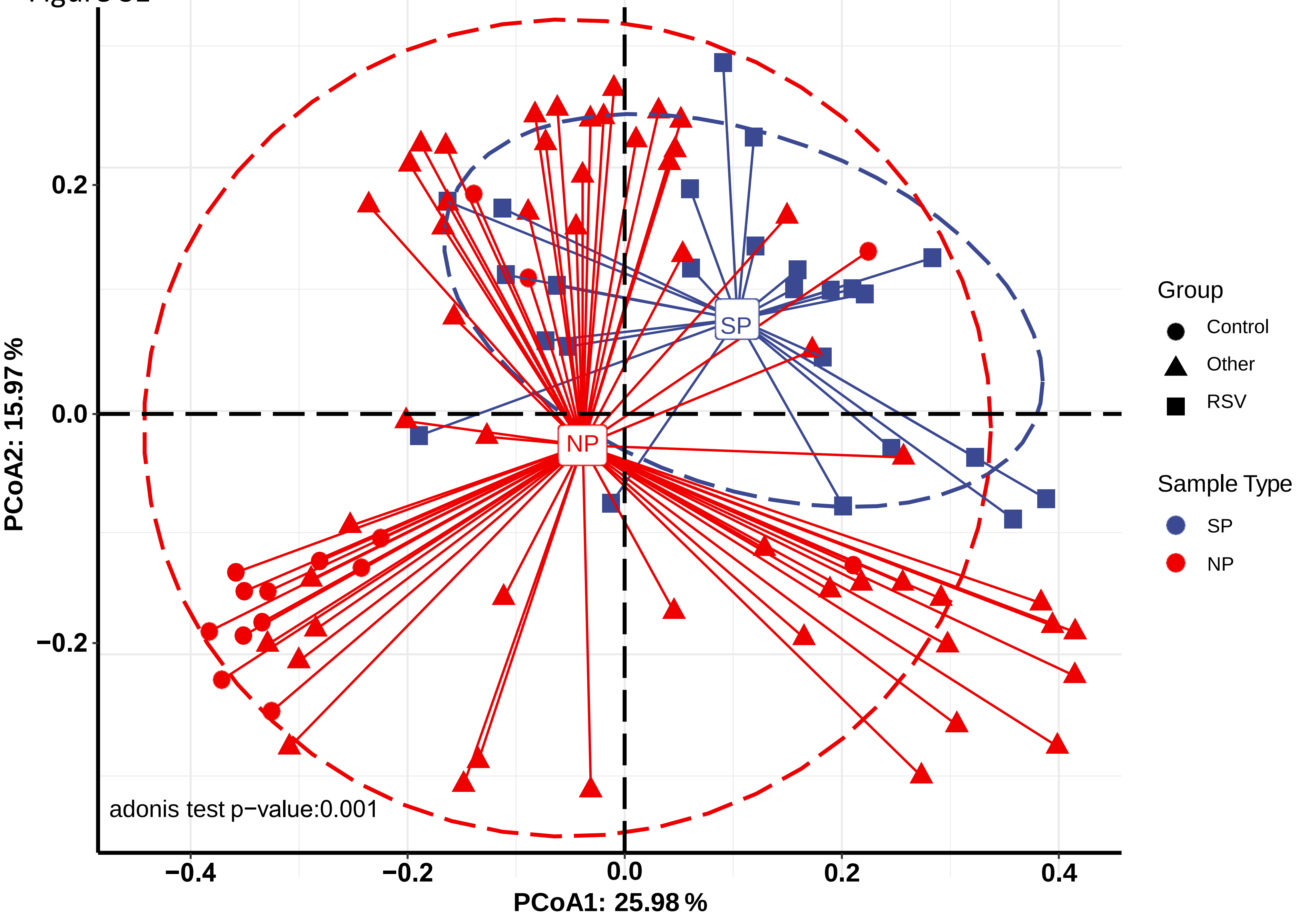

PCoA2: 15.97 %
PCoA1: 25.98 %
0.2
0.0
−0.2
−0.4
−0.2
0.0
0.2
0.4
SP
NP
adonis test p−value:0.001
Group
Control
Other
RSV
Sample Type
SP
NP

Figure S2

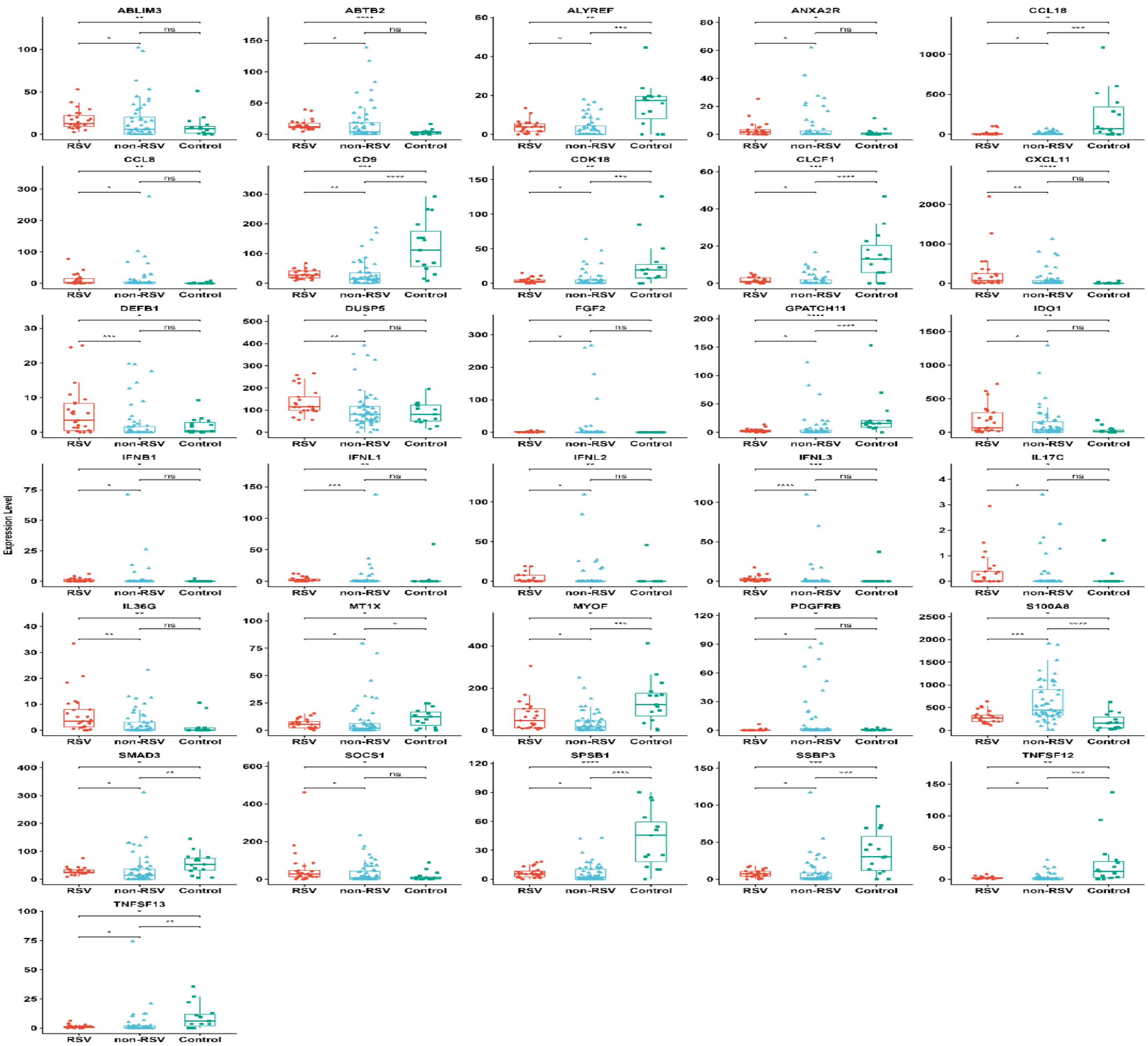
ABLIM3
ABTB2
ALYREF
ANXA2R
CCL18
CCL8
CD9
CDK18
CLCF1
CXCL11
DEFB1
DUSP5
FGF2
GPATCH11
IDO1
IFNB1
IFNL1
IFNL2
IFNL3
IL17C
IL36G
MT1X
MYOF
PDGFRB
S100A8
SMAD3
SOCS1
SPSB1
SSBP3
TNFSF12
TNFSF13
Expression Level
RSV
non-RSV
Control
ns

Figure S3

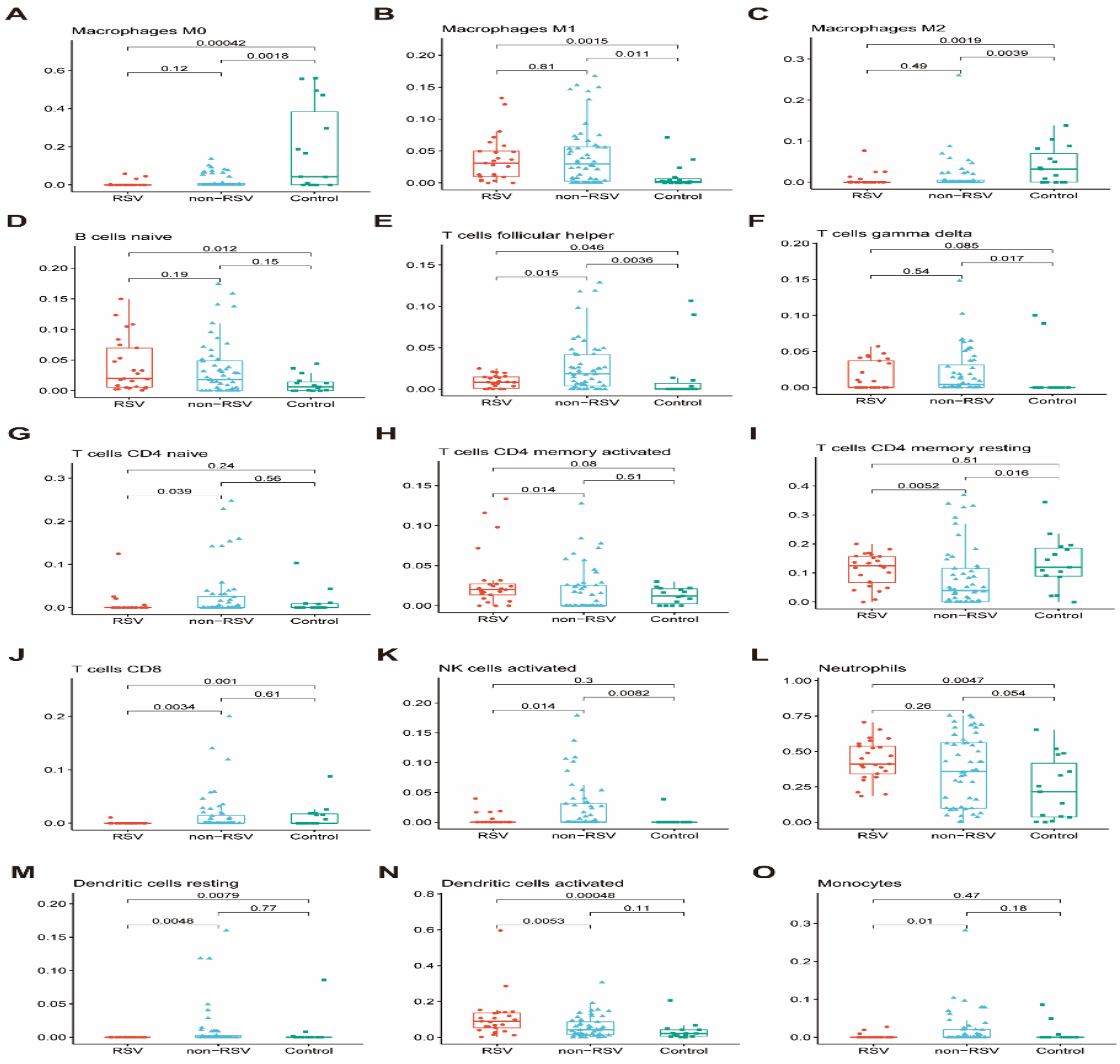
A
Macrophages M0
0.00042
0.0018
0.12
B
Macrophages M1
0.0015
0.011
0.81
C
Macrophages M2
0.0019
0.0039
0.49
D
B cells naive
0.012
0.15
0.19
E
T cells follicular helper
0.046
0.0036
0.015
F
T cells gamma delta
0.085
0.017
0.54
G
T cells CD4 naive
0.24
0.56
0.039
H
T cells CD4 memory activated
0.08
0.51
0.014
I
T cells CD4 memory resting
0.51
0.016
0.0052
J
T cells CD8
0.001
0.61
0.0034
K
NK cells activated
0.3
0.0082
0.014
L
Neutrophils
0.0047
0.054
0.26
M
Dendritic cells resting
0.0079
0.77
0.0048
N
Dendritic cells activated
0.00048
0.11
0.0053
O
Monocytes
0.47
0.18
0.01
RSV
non−RSV
Control

Figure S4A

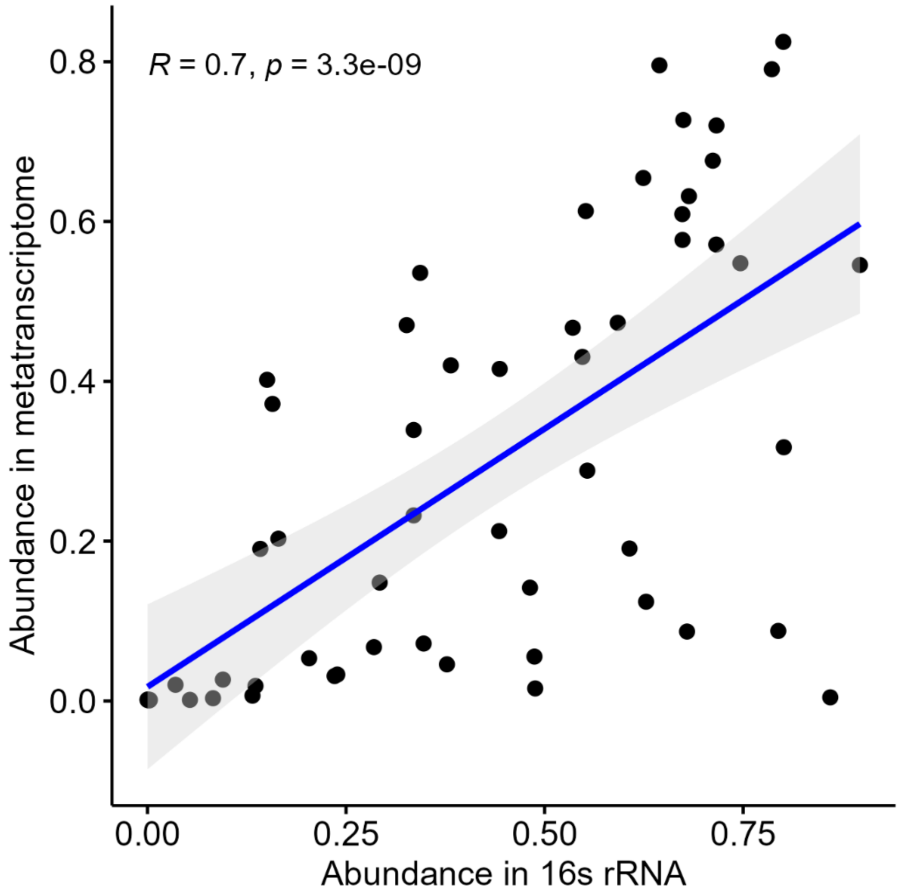

R = 0.7, p = 3.3e-09
0.8
0.6
0.4
0.2
0.0
Abundance in metatranscriptome
0.00
0.25
0.50
0.75
Abundance in 16s rRNA

Figure S4B

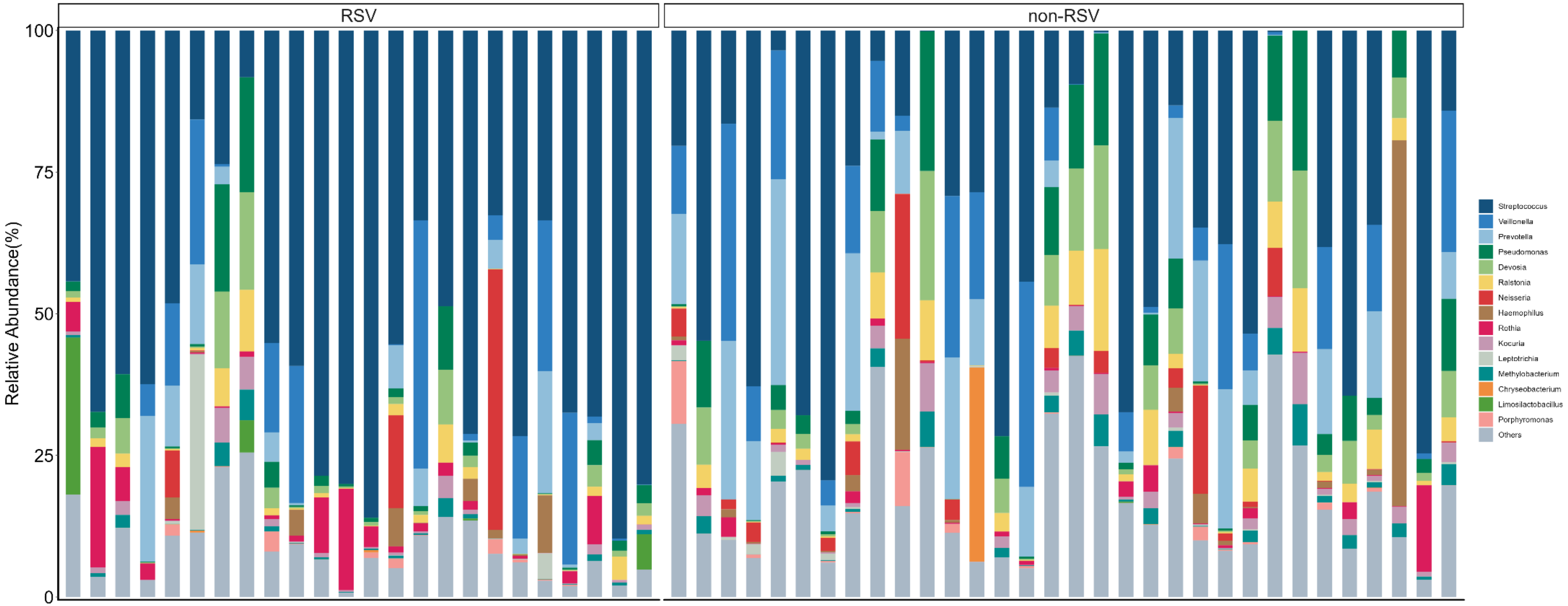
RSV
non-RSV
Relative Abundance(%)
100
75
50
25
0
Streptococcus
Veillonella
Prevotella
Pseudomonas
Devosia
Ralstonia
Neisseria
Haemophilus
Rothia
Kocuria
Leptotrichia
Methylobacterium
Chryseobacterium
Limosilactobacillus
Porphyromonas
Others
